# Yet it Endures: The Persistence of Original Sin

**DOI:** 10.1007/s11079-022-09704-3

**Published:** 2022-12-21

**Authors:** Barry Eichengreen, Ricardo Hausmann, Ugo Panizza

**Affiliations:** 1 UC Berkeley, Berkeley, USA; 2 Harvard KSG, Cambridge, USA; 3 Geneva Graduate Institute, Geneva, Switzerland

**Keywords:** UC Berkele, H63, F34, C82

## Abstract

Notwithstanding announcements of progress, “international original sin” (the denomination of external debt in foreign currency) remains a persistent phenomenon in emerging markets. Although some middle-income countries have succeeded in developing markets in local-currency sovereign debt and attracting foreign investors, they continue to hedge their currency exposures through transactions with local pension funds and other resident investors. The result is to shift the locus of currency mismatches within emerging economies but not to eliminate them. Other countries have limited original sin by limiting external borrowing, passing up valuable investment opportunities in pursuit of stability. We document these trends, analyzing regional and global aggregates and national case studies. Our conclusion is that there remains a case for an international initiative to address currency risk in low- and middle-income economies so they can more fully exploit economic development opportunities.

## Introduction

Financial fragility in emerging markets has created much human suffering and economic pain.^1^ No wonder it has attracted research attention for decades, and especially since the Latin American debt crisis of the 1980s.

But simple explanations for financial fragility based on public overborrowing, which in turn assume that emerging markets are unable to contain fiscal spending, are at best incomplete, given that emerging markets encounter problems at levels of debt that are low by the standards of advanced countries. Further hypotheses are therefore needed to make sense of this phenomenon.

A first hypothesis is that emerging markets are burdened by more serious credibility problems than their advanced-country counterparts. If for whatever reason their commitment to fiscal sustainability is less credible, markets will be quicker to anticipate defaults and inflation surprises; hence such countries will face higher interest rates and more jittery investors. This hypothesis resonates with economists, from advanced countries in particular, who are predisposed to think that if emerging markets and developing countries get into trouble, those countries themselves must be at blame. But these hypotheses leave unanswered *why* emerging markets might be systematically less credible than advanced countries.^2^ This gap in argumentation in turn means that no guidance is provided on what they can do to escape their predicament (see Calvo 1988).

A second hypothesis is that emerging markets display fear of floating (Calvo and Reinhart 2002). Because they do not allow their exchange rates to fluctuate and thereby absorb shocks, they expose themselves to currency misalignments that result in exchange rate crises rather than smooth adjustment. Again, however, the question of what causes this this fear of floating is left hanging. One possible answer is the lack of credibility of their monetary policies resulting in high passthrough from exchange rate movements to prices, which renders more flexible exchange rates costly and undesirable. But this answer only leads back to the previous problem, namely that the lack of credibility is unexplained.

A third hypothesis focuses on currency mismatches on national balance sheets (Goldstein and Turner 2004). If a country has net foreign currency exposure, then it is more vulnerable to a crisis. Bad times will cause a currency depreciation that raises the cost of debt service, and that higher cost of debt service can result in further currency depreciation, in a vicious circle. But, again, this explanation goes only part way toward answering the question. Are currency mismatches just a policy mistake, or do they have a deeper cause?

Twenty-five years ago, we proposed another interpretation. We argued that the central cause of financial fragility is the inability of countries to denominate their foreign obligations in their own currency, a phenomenon that we termed “original sin.” We showed that the phenomenon is too widespread to be explained by unfavorable monetary and financial history. Certainly, many countries with a history of inflation and default suffer from original sin, but many others with relatively unblemished monetary and financial histories do so as well. We showed that the only robust correlate of a country's ability to borrow abroad in its own currency was its economic size. We speculated that this reflected the structure of investor demand (lack of appetite of international investors for obligations denominated in the currencies of small countries) and liquidity issues (that such obligations trade in thin markets) rather than the peculiarities of a country's own history.

We showed that the consequences were far-reaching. Inability to issue external debt in the country’s own currency is correlated with fear of floating, with the need to hold additional international reserves, with higher volatility of output and capital flows, and with lower credit ratings. Moreover, countries exhibiting this problem display procyclical rather than countercyclical fiscal and monetary policies. Hausmann et al. (2001) showed that fear of floating was robustly related to original sin, not to exchange rate passthrough. Flandreau and Sussman (2005) showed that original sin was highly persistent, having changed little since the middle of the nineteenth century.

A lot of liquidity has flowed under the bridge in the past quarter century, in both the "real" world of finance and academia. More governments have succeeded in issuing international debt in domestic currency, and still others have seen greater participation of foreign investors in their domestic local-currency debt markets (Carstens and Shin 2019; Hofmann et al. 2020, 2021; Shin and Von Peter 2022). These observations in turn suggest important research questions. For example, were these changes due to much improved liquidity in those markets after the adoption of social security reforms that facilitated the establishment and development of pension funds? Did more credible inflation targeting regimes reduce one-sided currency risks and facilitate foreign participation? Hausmann and Panizza (2011) assessed this issue a bit over a decade ago; they showed that there had been modest progress in reducing original sin and considerable progress in implementing countercyclical macroeconomic policies, thanks to a reduction of currency mismatches and also to the accumulation of international reserves.

In this paper, we assess the changes in original sin over the quarter century since we originally visited this topic. We are “unrepentant.” We show that in the majority of developing countries the pattern persists. Emerging-market governments (defined here as middle-income countries with access to international financial markets and which tend to be included in the leading emerging market bond indices) have made progress in placing external debt securities denominated in their own national currencies with international investors. Low-income countries, in contrast, have made little such progress. Hence the case for currency hedges for low-income countries. Moreover, for emerging markets and developing countries overall, local currency issuance peaked in 2008–2011, a period of rapid growth in the emerging-market world and when advanced-country central banks, responding to the global financial crisis, cut interest rates to zero, encouraging investors to migrate toward bonds of other countries denominated in other currencies. Our data show that a nonnegligible share of that progress was reversed subsequently.

In terms of determinants, our previous results carry over. Aggregate country size remains the most robust determinant of the extent of original sin. It remains considerably more difficult for small as opposed to large countries to issue domestic-currency debt and sell it to foreign investors. Evidence of weak policies and institutions as a factor behind this pattern is more limited. This suggests that structural problems in international financial markets, and not simply current and past problems in individual emerging markets and developing countries, play a role in the phenomenon. And that conclusion in turn suggests also that there is a role for systemic solutions, such as ramping up international mechanisms that might enable such countries to better hedge their currency exposures.

## Literature

A large literature has grown up around the issues of currency mismatches, debt denomination, and their causes and consequences for emerging markets. Broadly speaking, this literature can be divided into three categories: the causes of mismatches and foreign-currency debt denomination, consequences for economic and financial stability, and trends in issuance.

The literature on currency mismatches goes back to Eichengreen and Hausmann (1999), Eichengreen et al. (2007) and Goldstein and Turner (2004). Eichengreen and coauthors emphasized the difficulty that developing countries, small developing countries in particular, have in issuing international debt denominated in their own currencies, and the risks of foreign currency issuance. Goldstein and Turner for their part pointed to policy measures that might limit foreign currency issuance and its associated perils.

More recently, Hale et al. (2020) point to issuers’ inflation history and global financial conditions as factors limiting the capacity to issue domestic currency debt. Han (2022) emphasizes the importance of local capital market depth and development. Romero et al. (2021) and Arslanalp et al. (2020) similarly emphasize the importance of global economic conditions, in their case highlighting the role of tracker funds that follow the JP Morgan emerging-market index. Ottonello and Perez (2019a, b) and Bassetto and Galli (2019) emphasize inflation risk. Engel and Park (2018) show in a theoretical model, countries with less credible monetary policies borrow mainly in foreign currency as a substitute for monetary credibility. Lee (2022) provides evidence that exchange-rate volatility, which is related to monetary policy volatility but can also depend on other factors, encourages foreign-currency issuance by emerging market sovereigns. Gegenfurtner (2021) highlights the importance of country size as a determinant of the currency composition of sovereign debt, small countries being reliant on foreign-currency issuance. Ballard-Rosa et al. (2022) emphasize the political aspect: analyzing data on 240,000 primary market transactions, they conclude that right-wing governments choose foreign currency denomination as a way of mitigating currency risk (tying the central bank’s hands), while left-wing governments prefer domestic-currency denomination, which facilitates more flexible use of monetary policy.

On the perils, a number of studies provide further evidence that adverse terms-of-trade shocks, economic contractions and exchange-rate depreciation can make foreign-currency-denominated debt unserviceable, curtail market access, lead to default, and cause serious problems for domestic economic and financial systems. Carstens and Shin (2019) and Shin and von Peter (2022) caution that domestic-currency denomination does not necessarily free emerging markets from these risks. They conclude that the currency risk borne by foreign investors renders their portfolio decisions and, by implication, emerging markets themselves more sensitive to global economic conditions. The irony, they conclude, is that local-currency bonds are sensitive to global economic conditions, while foreign-currency-denominated bonds are sensitive, via default risk, to local economic conditions – the opposite of what has long been argued.

A substantial literature highlights and documents the resulting procyclical behavior of foreign investors in local-currency bond markets. Fonay (2022) analyzes aggregate data for a panel of seven Latin American countries. Regressing spreads on foreign investor shares and controls, he shows that foreign holdings raise yield spreads when the local currency is expected to depreciate, but reduce spreads when the local currency is expected to appreciate. Bertaut et al. (2020) report complementary evidence, confirming that dedicated EM mutual funds behave more procyclically than other investors. Hofmann et al. (2022) concur that local-currency denomination is no panacea for countries seeking to borrow abroad. Currency mismatch is simply shifted to global lenders, who are apt to react strongly to currency fluctuations. Tighter global financial conditions can constrain bond purchases by such lenders, transmitting the financial tightening to even “virtuous” emerging markets. Carstens and Shin (2019) similarly conclude that local currency foreign borrowing has just shifted the locus of the currency mismatch to foreign lenders. The fundamental problem, they argue, is the shallowness of domestic financial markets and absence of a sizable domestic investor base, which together make it difficult for international lenders to hedge currency risk.

A considerable literature documents the growth of local currency bond markets in middle-income emerging markets in recent years, while at the same time emphasizing the heavy participation of foreign investors and the ongoing difficulty of the corporate sector, in particular, of borrowing abroad in the local currency. An early study of developments since the turn of the century was Burger et al. (2012), who documented the growth of local currency bond markets. They found that their growth was greatest in countries with improved macroeconomic stability and strengthened creditor rights. An official follow-up was IMF/World Bank (2016), which noted the continuing importance of foreign investors in those local currency markets.

The subsequent literature divides between country-specific and cross-country analyses. Rossini et al. (2020) study the case of Peru. Factors contributing to the development of local-currency bond markets include inflation targeting, creation of a secondary market for government bonds, solid macroeconomic fundamentals, improved credit rating and abundant global liquidity following the Global Financial Crisis. The main market participants are nonresident investors, pension funds and domestic banks. The participation of nonresident investors varies over time and is associated with the commodity price and capital flow cycle. Romero et al. (2021) consider the case of Colombia. They document increased purchases of local-currency bonds by foreign investors. This has increased market liquidity and reduced borrowing costs but also heightened the sensitivity of local financial conditions to global factors and to fluctuations in the Emerging Market Bond Index (EMBI) in particular.

In an example of the cross-section or panel approach, Shin and von Peter (2022) use published BIS government bond statistics for 25 emerging markets to document the decline in emerging market sovereigns’ reliance on foreign-currency denominated bonds.^3^ Du and Schreger (2017) considered 14 larger emerging markets, showing that their sovereigns increasingly borrow from foreigners in local currencies but that the private sector continues to borrow in foreign currency. As we show below, in assessing these findings it is important to note that patterns discerned for larger middle-income countries may not carry over to smaller low-income countries.

Vankatesh and Hiremath (2021) construct a measure of currency mismatch for 22 emerging markets over the period 2008–2018: they find that currency mismatches persist. These are positively associated with country size, inflation volatility and exchange rate depreciation. They are negatively associated with limited debt exposure, good monetary and fiscal policies, institutional quality and export openness. Aizenman et al.  (2020) consider sovereign bond issuance in 8 major emerging markets over the period 1970–2018. They show that EM local currency bonds are smaller in size, shorter in maturity than foreign currency bonds. They conclude that emerging markets are more likely to issue local currency bonds when currencies are strong and if they are inflation targeters. Some of these relationships weaken, however, following the Global Financial Crisis.

Mizen et al. (2021) focus in on the continued difficulty experienced by emerging market corporates attempting to borrow long term in local currency. They attribute difficulty this to market underdevelopment (lack of local market liquidity and investor diversity) and lack of experience (showing that such borrowing becomes easier for “seasoned” borrowers). Han (2022) reaches similar conclusions. Using hand collected data for 16 emerging markets, he traces changes in the currency composition and instruments (equity, debt, FDI) of emerging markets’ external labilities. He documents the declining exposure to foreign currency liabilities overall, while finding that corporate sector still has sizeable currency mismatch.

COVID-19 is a kind of stress test or natural experiment for analyzing the robustness of local currency bond markets. A recent literature thus considers how emerging market issuance responded to the COVID crisis, when many governments borrowed in an effort to buffer the economic and public-health impact. Shin (2021) shows that spreads on foreign-currency-denominated bonds rose only briefly at the outset of the crisis, but that those on domestic currency bonds rose and remained elevated. Venkatesh and Hiremath (2021) find that currency mismatches in fact increased in a number of Latin American and Central European emerging markets following the outbreak of COVID, as governments were forced to resort to foreign currency funding. Financial Stability Board (2022) shows that a number of emerging markets experienced sharp outflows from foreign-currency bond markets at the outset of the crisis, and that turbulence there spilled over to local markets. This again highlights the procyclicality of the purchases and sales of dedicated EM investment funds, consistent with work by Romero et al. (2021) and Arslanalp et al. (2020) cited above.

A more limited literature analyzes the determinants of “domestic original sin,” defined as the share of government bonds held by residents that are denominated in foreign currencies, have long maturities and bear fixed interest rate. Mehl and Reynaud (2005) compile a dataset on this for 33 countries from national sources. They find that domestic original sin is severe when inflation is high, the debt-service-to-GDP ratio is elevated, the yield curve is inverted, and the domestic investor base is narrow. They conclude that countries need to attack this problem on multiple fronts, adopting sound and stable macroeconomic policies, offering attractive long-term yield, and widening the domestic investor base. Borensztein et al. (2004) discuss select determinants of domestic debt composition in emerging economies, emphasizing the role of macroeconomic policies and the size of the investor base, but do not offer a formal statistical analysis. Borensztein et al. (2008) reach similar conclusions by focusing on a group of Latin American countries.

Finally, a small literature considers currency hedging for international portfolios. Schittman (2010) shows that hedging of currency exposure in single- and multi-country bond portfolios can significantly reduce the volatility of returns for Germany, Japanese, British and American investors. Alfaro et al. (2021) analyze foreign exchange derivative transactions in Chile. They show that financial hedging is used mainly by large firms and that firms pay significantly larger premiums for longer maturity contracts. In the case of Peru, Rossini et al. (2020) show that nonresident investors hedge their domestic currency sovereign bond) position partially with nonderivative forwards, a practice which has also contributed to the development of the FX derivatives market.

## Measuring Original Sin

Eichengreen et al. (2005a, b, 2007) propose two measures of international original sin based on security issuance data.^4^ The first focuses on the share of external bonds in domestic currency issued by country $i$ in year $t$. Formally:

$${OSIN1}_{i,t}=1-\frac{Securities\;Issued\;by\;Country\;i\;in\;Currency\;i}{Securities\;Issued\;by\;Country\;i}$$

**Fig. 1:**
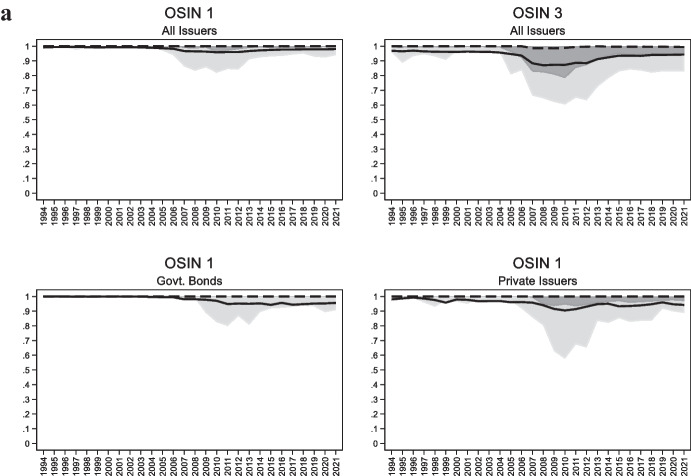

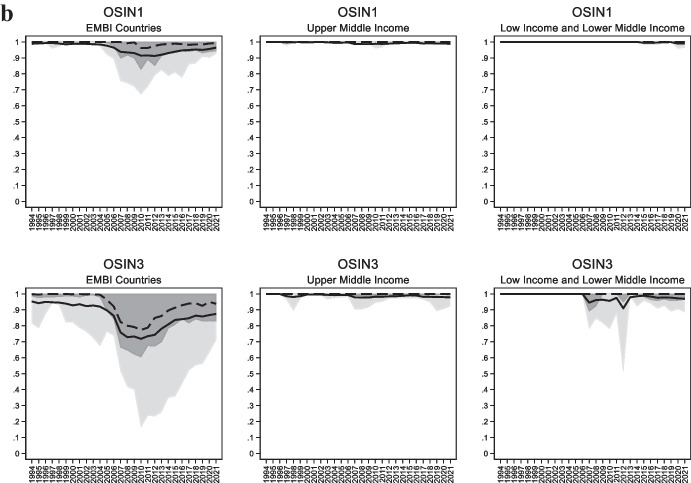
**a** Evolution of Original Sin (1994–2021). The four panels plot the evolution of the cross-country median (dashed line), mean (solid line), 25^th^ percentile (dark shaded area), and 90^th^ percentile (light shaded area) of various indexes of original sin for a sample of 85 emerging and developing economies. The top panels use all bonds and notes and the bottom panels only use bond and notes issued by governments (bottom left panel) and private issuers (right panel). **b** Evolution of Original Sin by Country Groups (1994–2021). The six panels plot the evolution of the cross-country median (dashed line), mean (solid line), 25^th^ percentile (dark shaded area), and 90^th^ percentile (light shaded area) of OSIN1 (top panels) and OSIN 3 (bottom panel) for countries that are part of the EMBI index (first column), upper middle-income countries that are not part of the EMBI index (middle column) and low and lower middle-income countries (last column)

The top left panel of Fig. 1 describes the evolution of *OSIN 1* for an unbalanced panel of 85 developing and emerging economies. It shows that the median country issues 100% of its debt in foreign currency and that this has not changed over the period under observation, 1994 to the present. When considering average values, we find that the share of foreign currency debt ranges between 95 and 99%. Even the country at the 25^th^ percentile of the distribution was never able to issue more that 4% of its external bonds in domestic currency. If we focus on the bottom 10^th^ percentile of the distribution, we find a small sample of countries that, starting in 2007, were able to issue between 10 and 20% of their external bonds in domestic currency. The index of original sin for this part of the distribution bottomed at 82% in 2010 and then went back to 92% in 2013. The bottom panels of the Fig. 1 show that private issuers have lower levels of original sin than official issuers.

The *OSIN 1* index in Fig. 1 has at least three limitations. First, it does not recognize that countries use derivative instruments to hedge currency risk. Second, it is based on securities and does not include other types of external debt. Third, data on international debt securities might not keep track of bonds issued domestically but bought by non-residents.

Eichengreen et al. (2005a, b, 2007) address the first issue by recognizing that a country is more likely to be able to hedge its foreign currency position if there are market participants willing to take the currency risk. Hence, the total amount of securities issued by country $i$ in currency $i$ is not the relevant variable; the important thing is the total amount of securities issued in currency $i$, no matter who issued them. Eichengreen et al. (2005a, b, 2007) also recognize that a country cannot hedge more than what it issues. Hence, they define an alternative index of original sin as:

$${OSIN\;3}_{i,t}=Max\left(1-\frac{Securities\;Issued\;in\;Currency\;i}{Securities\;Issued\;by\;Country\;i},\;0\right)$$

The top right panel of Fig. 1a shows that, like the first index, the median value of OSIN 3 has remained close to 1 throughout the sample.^5^ There are no international issuances in the domestic currency for the typical emerging and developing economy. Focusing on the average of *OSIN 3*, we find values which are often above 90%. On average, there was a small reduction in original sin in 2007–2012, a period of rapid growth and extensive market access for many emerging economies. But even in this period the index never went below 87% (over 2015–21, the index went back to 94%). Even the country at the bottom 10^th^ percentile of the distribution never went below 63%.

**Fig. 2:**
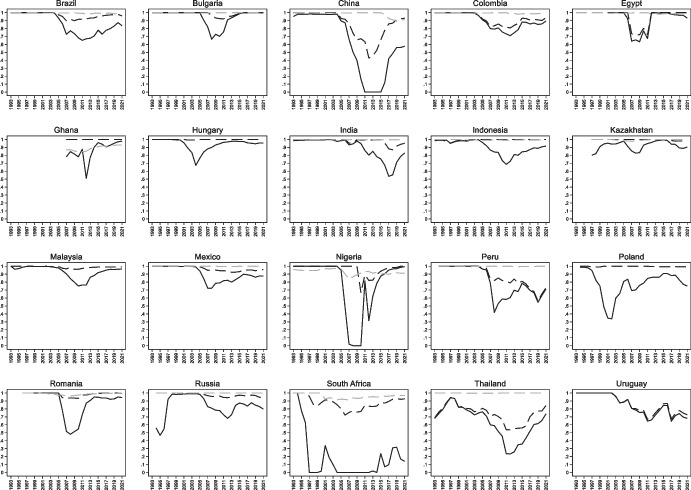
Evolution of Original Sin in Selected Economies. The figures plot the evolution of OSIN 1 computed with BIS data (dashed black line) OSIN 1 computed with World Bank data (dashed grey line), and OSIN 3 (solid black line) in all emerging and developing economies which, at any point in time had values of OSIN 3 below 0.8

In our sample, 20 countries had a value of *OSIN 3* below 0.8 at any point in time. Figure 2 plots the country-by-country evolution *OSIN 1* and *OSIN 3* for these twenty countries. Here two things are worth noting.

First, more than half of the countries described in Fig. 2 have *OSIN 1* indexes that never go below 0.9.^6^ Most cases of “redemption” from original sin are driven by *OSIN 3*. This indicates that in these countries (which includes all Eastern and Central European countries of Fig. 2, and all Asian countries except China and Thailand), redemption from original sin is entirely or almost entirely driven by issuance by non-residents.

Second, for most countries local currency issuance peaked around 2008–11. Peru and Uruguay are the only countries in which the redemption process did not reverse in the last 8–10 years. (We discuss these cases further below.)

Another way of cutting the data is between emerging markets included in the EMBI index, other upper-middle-income emerging markets, and low- and lower-middle-income economies. We show *OSIN 1* and *OSIN 3* for the three groups separately in Fig. 1b. We see that the decline in original sin around the middle of the period was concentrated in EMBI countries. In contrast, there is very little movement in either *OSIN 1* or *OSIN 3* for other middle-income countries and low-income countries.

The second issue is that these indexes only use securities. Building an index of original sin that also includes bank loans is complicated by the fact that only limited data exist on the currency composition of these obligations. One possibility is using data from the World Bank International Debt Statistics (IDS) to approximate the currency composition of all public and publicly guaranteed (PPG) debt. IDS reports data on the share of PPG external debt denominated in US dollars, euros (before the creation of the euro, IDS reported data for debt denominated in German marks and French francs), Japanese yen, British pounds, Swiss francs, SDRs, multiple currencies, and “other currencies.” By assuming that all PPG debt in “other currencies” is denominated in domestic currency, we can obtain a lower bound for an index of original sin that includes both debt securities and loans (but only for PPG debt).^7^ These values should be treated as a lower bound because some countries issue debt in a currency which is neither their own nor one of the currencies listed by the World Bank. For instance, several countries in southern Africa borrow in South Africa rand and Chinese RMB. Several countries in Central and East Asia borrow in RMB and Russian rubles.^8^

**Fig. 3:**
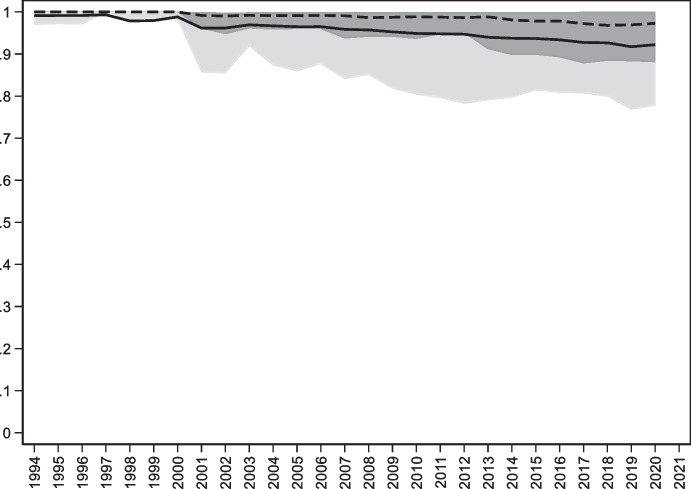
Original Sin with World Bank data (1994–2021). This figure plots the evolution of the cross-country median (dashed line), mean (solid line), 25^th^ percentile (dark shaded area), and 90^th^ percentile (light shaded area) of OSIN 1 computed using World Bank IDS data for the stick of public and publicly guaranteed bonds under the assumption that the “other currency category” refers to domestic currency

Figure 3 shows the evolution of original sin based on World Bank IDS data. Trends are similar to those of Fig. 1 with one notable exception: World Bank data suggest that redemption from original sin is more enduring than that indicated by BIS data. One explanation for this is the already mentioned fact that we are capturing a lower bound on original sin. For instance, several African countries are now borrowing heavily in Chinese RMB, but since World Bank data put RMB loans in the “other currency” category, we classify these loans as being in domestic currency. Another explanation is that while the index of original sin that uses securities is based on bond issuance, the index that uses World Bank data is based on outstanding bonds and loans. As it based on stocks rather than flows, this index is naturally more persistent.

**Fig. 4:**
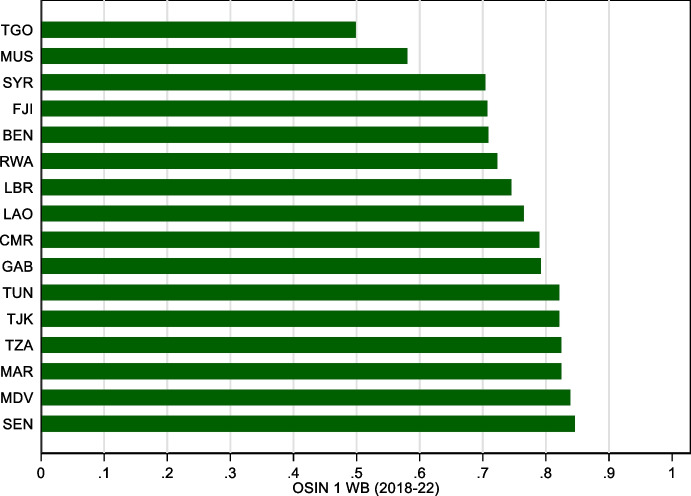
Original Sin with World Bank data (Selected economies 2018–22). This figure plots the average value for 2018–22 of OSIN 1 computed using World Bank IDS data for all countries with values of the index below 0.85

Although the overall trend of the original sin index computed with World Bank data on total PPG debt is similar to that of the index computed with BIS securities, the country-level correlation between the two indexes is basically zero. This is partly due to the abovementioned loans in RMB, rand and rubles. By misclassifying these loans as local, the index based on World Bank data leads to low values for several countries that, we suspect, are not able to borrow in local currency (Fig. 4).

The official definition of external debt focuses on the residence of the creditor. The *External Debt Statistics: Guide for Compilers and Users* jointly published by the BIS, Eurostat, IMF, OECD, Paris Club, UNCTAD and the World Bank states that: “Gross external debt, at any given time, is the outstanding amount of those actual current, and not contingent, liabilities that require payment(s) of principal and/or interest by the debtor at some point(s) in the future and that are owed to non-residents by residents of an economy.” This official definition makes sense from a theoretical standpoint, insofar as it focuses on the transfer of resources between residents and non-residents and measures the extent of international risk sharing. However, this residency-based definition is difficult to apply to tradable securities. Only a small number of emerging and advanced economies are able to track the residence of buyers of their securities in the primary market and to follow what happens in the secondary market. They do so by running periodic surveys or because the central bank works closely with the custodians of the securities. But most developing countries do not have tools for identifying the ultimate holders of their bonds.

World Bank data on external debt are based on the Bank’s Debtor Reporting System (DRS). This system utilizes data collected by issuing governments. If those governments do not report information on domestically issued bonds held by non-residents, then IDS data will overestimate original sin. Consider, for instance, the light grey lines of Fig. 2. They show that for most countries (e.g., Colombia, Peru, South Africa, and Thailand) the index built using World Bank data remained close to one, even though international securities data from the BIS report a substantial amount of local currency-denominated securities. We suspect that these are securities issued in local markets and bought by non-residents, and that these securities are not included in the World Bank’s International Debt Statistics.

Yet another source for measuring original sin is the Arslanalp and Tsuda (2014) dataset. This dataset tracks the composition, by currency and holders, of government securities issued by up to 22 emerging markets over the period 2004–21. The authors draw on the IMF and World Bank’s *Quarterly External Debt Statistics*, the IMF’s *Government Finance Statistics* and *International Financial Statistics*, and national sources. The resulting data set is rich, but two issues with it are limited coverage both in terms of time (it only starts in 2004) and number of countries, and the fact that it only provides information on government bonds. In practice the countries included are larger middle-income economies, whose distinctive behavior we flagged in Fig. 1a.

Arslanalp and Tsuda data can be used to compute two measures of ability to borrow in a country’s own currency. One is foreign currency government debt held by non-residents as a share of total government debt held by non-residents. The second is the share of foreign currency government debt as a share of total public debt. The first measure is comparable to the *OSIN 1* measure described above. The second allows us to assess the propensity of residents to buy domestic currency debt (which can be taken as capturing domestic original sin, defined as the share of foreign currency central government bonds held by residents). These measures can thus be used to assess overall foreign investors participation in the government bond market.

**Fig. 5:**
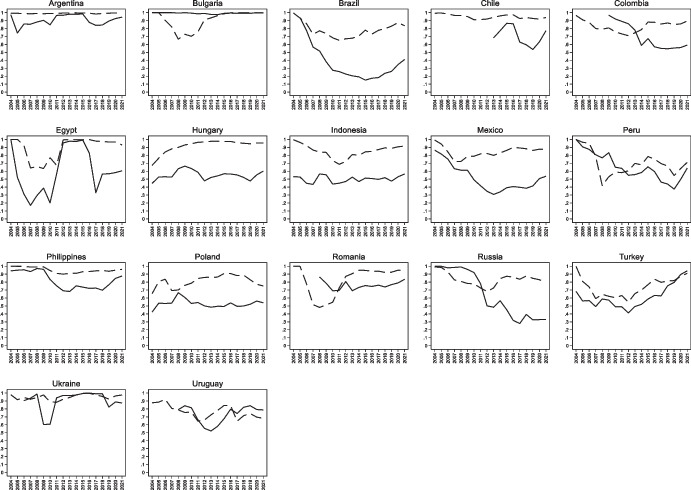
International Original Sin using BIS Securities versus Arslanalp and Tsuda Data for Central Government Debt. The figures plot OSIN3 computed with BIS data (dashed black line) and the share of foreign currency debt for central government securities held by non-residents (solid black line) computed using Arslanalp and Tsuda data

Figure 5 compares the country-by-country evolution of *OSIN3* with that of the original sin index computed using the Arslanalp-Tsuda data. While *OSIN 3* tends to have higher values (as before), for most countries the two indexes move together. There is, in fact, a close correlation between the two indexes both across and within countries of (column 1 of Table 1).

**Table 1:** Correlation among different index of original sin and between original sin and the share of external debt held by non-residents

	(1)	(2)	(3)	(4)	(5)
	OSIN AT	OSIN AT	OSIN3	OSIN AT	OSIN3
	**Pooled OLS**				
OSIN3	0.678***				
	(5.136)				
DSIN		0.779***	0.147***		
		(12.484)	(3.798)		
NRES				0.535***	0.268***
				(5.952)	(3.593)
Constant	0.0291	0.480***	0.830***	0.430***	0.709***
	(0.257)	(24.242)	(99.520)	(10.047)	(26.062)
R-squared	0.087	0.241	0.047	0.094	0.041
	**Country FE**				
OSIN3	0.455***				
	(2.630)				
DSIN		0.259	0.301***		
		(1.018)	(3.047)		
Ext Debt				-0.384	0.0509
				(1.714)	(0.822)
Constant	0.219	0.563***	0.805***	0.730***	0.770***
	(1.490)	(13.799)	(50.949)	(9.959)	(44.253)
N. Obs	312	310	310	310	385
R2	0.093	0.024	0.079	0.055	0.002
N. Countries	19	19	19	19	22

This table shows the correlation between the index of international original sin computed using Arslanalp and Tsuda data (OSIN AT) and OSIN 3 and the correlation between these two indexes of original sin and each of the index of domestic original sin computed using Arslanalp and Tsuda data (DSIN), and the share of central government bonds held by non-residents (NRES) computed with Arslanalp and Tsuda data. The top panel estimates the correlation using pooled OLS and the bottom panel includes country fixed effects

Robust t statistics in parenthesis

^*^p < 0.10; ^**^p < 0.05; ^***^p < 0.01

**Fig. 6:**
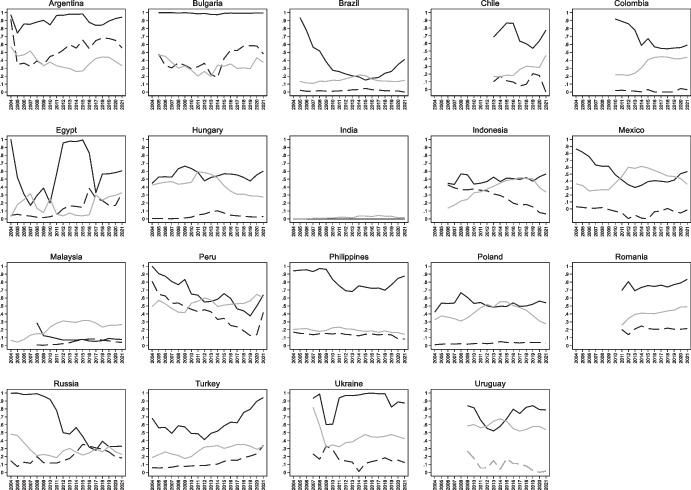
International Original Sin, Domestic Original Sin, and External Debt (Arslanalp and Tsuda Data). This figure plots the share of foreign currency debt for central government securities held by non-residents (solid black line) the share of foreign currency debt for central government securities held by residents (solid gray line), and the share of central government securities held by non-residents

Figures 5 and 6 plot the country-by-country evolution of the original sin index computed with Arslanalp-Tsuda data, together with the domestic original sin index and the external debt share constructed from the same data. While there is a strong cross-country correlation between the Arslanalp-Tsuda-based original sin index and each of domestic original sin and external debt share (columns 2 and 4 of the top panel of Table 1), the within-country correlation of these variables is not statistically significant (columns 2 and 4 of the bottom panel of Table 1). There is, instead, a significant cross- and within-country correlation between *OSIN 3* and domestic original sin (column 3 of Table 1). The correlation between *OSIN 3* and the external debt share is instead insignificant when we only consider within-country variation (column 5 of bottom panel of Table 1).

## Drivers of Original Sin

A key message of Eichengreen et al. (2005a, b, 2007) was that original sin is not associated with standard measures of policies and institutions; the only variable strongly associated with it is country size, as proxied by aggregate GDP. In what follows we estimate a set of regressions aimed at checking if this result still holds when we use our new updated data.

For three reasons, we focus only on emerging and developing countries (unlike Eichengreen et al. 2005a, b, 2007, which considered the universe of countries). First, policy discussion has focused on this set of countries. Second, we want to be sure that our results are not simply driven by comparing rich and poor countries. Third, many advanced economies now belong to the euro area, and defining original sin for members of a common currency area is not straightforward (see De Grauwe 2012; Dell’Erba et al. 2013).

We start by regressing *OSIN 3* on the same country characteristics as in Eichengreen et al. (2005a, b, 2007) for different period of time. Specifically, we compute averages for three five-year periods (2005–2009, 2010–14, and 2015–19) and then also for the COVID-19 period (2020–21). We regress original sin on the log of income per capita, the debt to GDP ratio, log of GDP, credit to the private sector scaled by GDP, an index of rule of law, log inflation, and trade openness.

Columns-1–4 of Table 2 show that aggregate GDP is always statistically significant at the 1% confidence level and that it is negatively correlated with original sin. This is the key result of Eichengreen et al. (2005a, b, 2007): larger countries are better able to borrow in own currency. Note that the negative correlation between original sin and country size is not driven by China and India, two large countries with low levels of original sin: all results are robust to dropping these two countries from the sample.

**Table 2:** The drivers of original sin

	(1)	(2)	(3)	(4)	(5)	(6)
	2005–09	2010–14	2015–19	2020–21	All years	All years
Ln(GDP PC)	0.010	0.009	0.012	-0.001	-0.037*	-0.219*
	(0.38)	(0.50)	(0.83)	(-0.09)	(-1.91)	(-1.96)
Debt/GDP	0.001	0.001	0.001	-0.001	0.001	0.001
	(0.29)	(1.43)	(1.00)	(-0.06)	(1.08)	(0.80)
Ln(GDP)	-0.043***	-0.053***	-0.034***	-0.027***	-0.041***	0.106
	(-4.83)	(-5.67)	(-4.80)	(-3.89)	(-4.75)	(1.39)
Pric Cr/GDP	-0.001	-0.003**	-0.002**	-0.001*	-0.001	0.001
	(-0.38)	(-2.14)	(-2.03)	(-1.82)	(-0.56)	(0.46)
RoL	-0.019	-0.013	-0.017	-0.035	-0.002	0.007
	(-0.64)	(-0.35)	(-0.60)	(-1.16)	(-0.08)	(0.24)
Ln(INFL)	0.030	-0.007	-0.005	-0.030**	0.001	-0.001
	(1.37)	(-0.37)	(-0.48)	(-2.16)	(0.04)	(-0.11)
OPEN	0.040	0.063	0.034	0.061	-0.004	0.002
	(0.82)	(1.14)	(0.70)	(1.52)	(-0.12)	(0.05)
Const	0.950***	1.118***	1.044***	1.172***	1.399***	2.275***
	(3.73)	(7.61)	(11.59)	(9.59)	(8.54)	(3.66)
R2	0.25	0.45	0.40	0.31	0.15	0.24
N Obs	60	76	80	68	1,211	1,211
Sample	Cross-Section (period average)				Panel (annual data)	
Country FE	No	No	No	No	No	Yes
Estimation	OLS	OLS	OLS	OLS	RE	FE

This table shows a set of regressions where the dependent variable is OSIN 3 and the explanatory variables are the log of GDP per capita (Ln(GDP PC)), public debt as a percentage of GDP (Debt/GDP), log of total GDP (Ln(GDP)), credit to the private sector over GDP (Pric Cr/GDP), and index of Rule of Law (RoL), the log of inflation (Ln(INFL)), and trade openness (OPEN). Columns 1–5 reports cross sectional estimates for period averages, and columns 6 and 7 report panel estimates that use annual data

Robust *t* statistics in parentheses

^*^p < 0.10; ^**^p < 0.05; ^***^p < 0.01

**Fig. 7:**
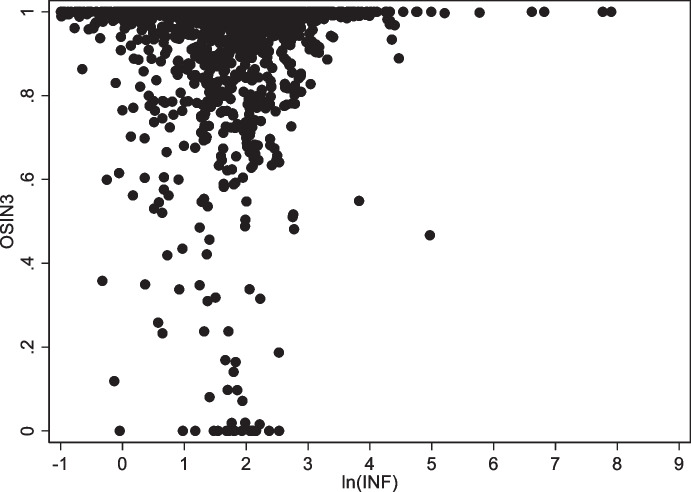
Original Sin and Inflation. This figure plots OSIN 3 (vertical axis) and log inflation (horizontal axis) using annual data for all emerging and developing economies

Two other variables are significantly correlated with original sin in at least some regressions. First, there is some evidence that countries with larger financial systems (as proxied by credit to the private sector over GDP) are more likely to issue abroad in their own currency. Second, there is a negative correlation in 2021 between inflation and original sin (Fig. 7).

**Fig. 8:**
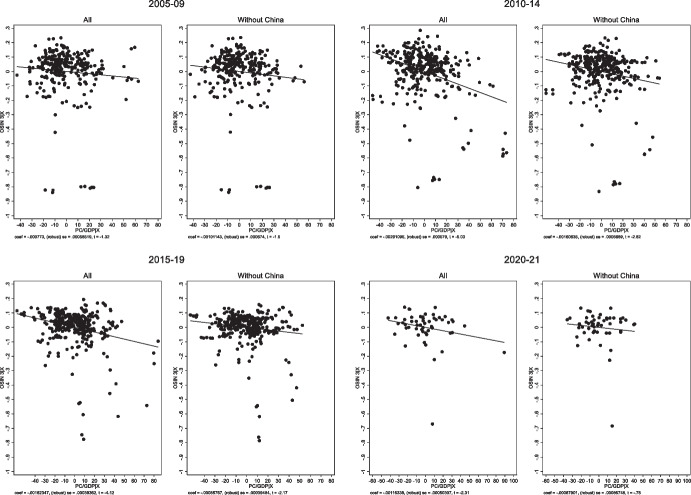
Original Sin and Credit to the Private Sector. The figures plot the partial correlation (controlling for all the variables included in Table 2) between OSIN 3 and credit to the private sector over GDP. The figures labelled “All” reproduce the regressions of columns 1–4 of Table 2, the figures labelled “without China” show the results of the same regressions when we estimate the model by dropping China from the sample

The first result is related to Tirole’s (2003) argument that lending in foreign currency is driven by a commitment problem that can potentially be solved by building a large domestic financial market. A large domestic market provides a domestic constituency of local-currency debt holders who will penalize a government that debases the currency and erodes the value of the debt. While Eichengreen et al. (2005b) found no statistically significant correlation between original sin and the size of the domestic financial sector, Table 1 finds some evidence pointing in this direction for the post 2010 period. However, Fig. 8 shows that this result is entirely driven by China, with its large (captive) market and extensive local-currency issuance. The correlation between credit to the private sector and original sin is never statistically significant when China is dropped from the sample.

The second result connecting inflation with local-currency issuance goes against the monetary-credibility hypothesis: it implies that countries with higher inflation are more likely to be able to borrow abroad in own currency. This result, however, is mostly driven by South Africa, and it is peculiar to the pandemic period. In general, there is no close relationship between inflation and original sin. Figure 7 shows that there is no country with high levels of inflation and low levels of original sin. This suggests that monetary credibility is necessary for escaping original sin but may not be sufficient. The figure shows that there are many countries with low levels of inflation and high levels of original sin.

Columns 5 and 6 of Table 2 use an unbalanced panel with annual observations for the entire 1995–2021 period. When we estimate the model without country fixed effects (column 5), we still find a strong negative correlation between aggregate economic size and original sin, and also a marginally significant negative correlation between GDP per capita and original sin. No other variable is statistically significant. When we include country fixed effects, most variables become insignificant (only GDP per capita remains marginally significant). This is not surprising, since country size is driven by population growth and the growth rate of per capita GDP. Population growth is a slow-moving variable that is highly correlated with country fixed effects. So, when we include country fixed effects the only remaining source of variability is changes in GDP per capita.

Table 3 shows the results when we use the index of original sin built using Arslanalp and Tsuda’s (2014) data on the currency composition of central government bonds held by non-residents (columns 1 and 2) and central government bonds held by residents (columns 3 and 4). When we focus on international original sin and do not include country fixed effects, we find the usual result that larger countries have lower levels of original sin. We also find that better institutions (as measured by the index of rule of law) are associated with lower levels of original sin (column 1 of Table 3). Somewhat surprisingly, income per capita is positively associated with original sin. This result is, however, entirely driven by India, which has both low income per capita relative to other countries in the sample and low levels of original sin.^9^ When we control for country fixed effects (column 2), we find that none of our variables of interest is significantly correlated with international original sin.

**Table 3:** The drivers of original sin (AT Data)

	(1)	(2)	(3)	(4)
	Non-Residents		Residents	
Ln(GDP PC)	0.128^**^	0.372	0.0729	0.189
	(2.12)	(1.07)	(0.0507)	(0.287)
Debt/GDP	-0.001	-0.000	0.0006	0.001
	(-0.79)	(-0.23)	(0.001)	(0.002)
Ln(GDP)	-0.194^***^	-0.409	-0.102***	-0.201
	(-5.09)	(-1.34)	(0.027)	(0.266)
Pric Cr/GDP	-0.003^*^	-0.003	0.0006	0.0007
	(-1.69)	(-1.30)	(0.001)	(0.001)
RoL	-0.235^**^	-0.329	-0.203***	-0.232***
	(-2.23)	(-1.09)	(0.046)	(0.060)
Ln(INFL)	0.030	0.032	0.0073	0.009
	(1.47)	(1.61)	(0.011)	(0.013)
OPEN	-0.137	-0.261	-0.164**	-0.214**
	(-1.32)	(-1.51)	(0.068)	(0.074)
Const	1.410^***^	0.806	0.640	0.283
	(3.07)	(0.58)	(0.516)	(1.087)
Obs	271	271	271	271
R^2^	.61	.25	.14	.15
Sample	Panel (annual data)			
Country FE	No	Yes	No	Yes
Model	RE	FE	RE	FE

This table shows a set of regressions where the dependent variable is the index of original sin computed using AT data and the explanatory variables are the log of GDP per capita (Ln(GDP PC)), public debt as a percentage of GDP (Debt/GDP), log of total GDP (Ln(GDP)), credit to the private sector over GDP (Pric Cr/GDP), and index of Rule of Law (RoL), the log of inflation (Ln(INFL)), and trade openness (OPEN)

*t* statistics in parentheses

^*^p < 0.10; ^**^p < 0.05; ^***^p < 0.01

When we consider the domestic component of original sin and do not control for country fixed effects, we obtain the usual result that larger countries have lower levels of original sin and, as in the case of international original sin, that institutional quality is negatively correlated with original sin (column 3, Table 3). Here, we also find a negative correlation between trade openness and original sin. When we include country fixed effects, we find that the correlation between country size and domestic original sin is no longer statistically significant, but that institutions and trade openness remain significant. While the result for trade openness is driven by Argentina (when we drop Argentina from the sample, openness is no longer significant in the fixed-effects regression), the result for rule of law is not driven by any particular country.

Summing up, updated data on overall international original sin and on the currency composition of government debt holdings of non-residents confirm Eichengreen et al. (2005b) original result: the only variable that is robustly correlated with the ability to borrow abroad in own currency is country size. There are a few countries that we discuss in detail Sect. 6 that have been able to escape from original sin, but these remain exceptional cases.

Data on domestic original sin also suggest that country size matters. In this case, however, we find an important role of institutions that is robust to controlling for country fixed effects.

Our regressions thus corroborate one of the most striking findings of Eichengreen et al.’s (2005b) original work. There is no robust correlations between fiscal fundamentals (as measured by inter alia the debt-to-GDP ratio) and monetary credibility (as measured by average inflation) on the one hand and the various measures of original sin on the other.

## Consequences of Original Sin

Another key message of Eichengreen et al. (2005a, 2007) was that original sin has important consequences for the conduct of macroeconomic policy. Among other things, Eichengreen et al. (2005a, 2007) show that countries that suffer from original sin are less likely to have a floating exchange rate. Hence such countries lack monetary independence in the presence of an open capital account. They also have lower credit ratings.

As in the previous section, we reassess these results using more recent data. For the exchange rate regime, we use data from Ilzetzki et al. (2019) and classify countries using an index that ranges between 1 (no separate legal tender or hard peg) and 13 (fully floating exchange rate regime). Given the ordinal nature of the dependent variables, we study the drivers of the exchange rate regime using an ordered logit model.

As before, we start with cross-sectional regressions based on 5-year averages and regress the index of exchange rate flexibility on original sin, the log of GDP per capita, external debt over GDP, public debt over GDP, and trade openness. Original sin is the only variable that is robustly correlated with the exchange rate regime. As Eichengreen et al. (2005a, 2007), we find that higher values of original sin are negatively associated with the presence of a floating exchange rate (column 1, Table 4).

**Table 4:** Original sin and Exchange rate regime (cross sectional regressions)

	(1)	(2)	(3)	(4)
	2005–09	2010–14	2015–19	2020–21
OSIN3	-6.467^***^	-6.488^***^	-8.816^***^	-13.900^***^
	(-3.44)	(-4.01)	(-3.94)	(-4.30)
Ln(GDP PC)	0.162	-0.213	-0.125	-0.345
	(0.51)	(-0.89)	(-0.51)	(-1.39)
Ext Debt/GDP	-0.012	-0.000	0.001	-0.002
	(-1.49)	(-0.05)	(0.20)	(-1.01)
Debt/GDP	0.014^*^	-0.002	-0.004	-0.001
	(1.90)	(-0.28)	(-0.52)	(-0.17)
OPEN	-0.060	-0.421	-0.032	0.895
	(-0.08)	(-0.61)	(-0.04)	(1.33)
Obs	61	77	77	76
Model	OLogit	OLogit	OLogit	OLogit

This table shows a set of regressions where the dependent variable is a numerical measure exchange rate regimes (higher values correspond to a more flexible exchange rate regime) and the explanatory variables are OSIN3, the log of GDP per capita (Ln(GDP PC)), public debt as a percentage of GDP (Debt/GDP), external debt over GDP (Ext Debt/GDP), and trade openness (OPEN)

*t* statistics in parentheses

^*^p < 0.10; ^**^p < 0.05; ^***^p < 0.01

When we use panel data without fixed effects, we still find that original sin is negatively correlated with the presence of a floating exchange rate, as are the level of external debt and trade openness. Surprisingly, we find that richer countries are less likely to have a floating exchange rate (column 1 of Table 5). The level of public debt, in contrast, does not seem to matter. To probe further, we split the sample into countries that are part of the EMBI index (column 2) and countries that are not (column 3). The correlation between exchange rate flexibility and income per capita is positive in the first group of countries and negative in the second. The opposite is true to trade openness. The regressions of columns 2 and 3 also indicate that the level of public debt is positively correlated with exchange rate flexibility for EMBI countries, whereas it does not matter for countries that do not belong to the index.

**Table 5:** Original sin and Exchange rate regime (Panel regressions)

	(1)	(2)	(3)	(4)	(5)	(6)
OSIN3	-5.999^***^	-2.159^***^	-17.674^***^	-1.443	-1.666	14.247
	(-15.22)	(-5.28)	(-10.56)	(-1.41)	(-1.57)	(0.85)
Ln(GDP PC)	-0.142^***^	0.915^***^	-0.447^***^	-0.831	-0.186	-3.151^**^
	(-2.68)	(8.74)	(-6.66)	(-1.32)	(-0.26)	(-2.32)
Ext Debt/GDP	-0.004^***^	-0.026^***^	-0.003^**^	-0.005	-0.001	-0.008
	(-3.23)	(-7.50)	(-2.11)	(-0.61)	(-0.04)	(-1.61)
Debt/GDP	0.002	0.025^***^	-0.001	0.019	0.051	-0.010
	(1.04)	(6.47)	(-0.29)	(1.10)	(1.61)	(-0.60)
OPEN	-0.399^***^	-1.212^***^	0.720^***^	-1.156	-2.469	4.245
	(-2.78)	(-5.23)	(3.73)	(-0.61)	(-1.48)	(1.09)
Obs	1492	603	889	852	475	377
R^2^	.05	.09	.05	.04	.08	.24
Sample	Panel (annual data)					
Countries	All	EMs	Non-EMs	All	EMs	Non-EMs
Country FE	No	No	No	Yes	Yes	Yes
Model	OLogit	OLogit	OLogit	OLogit	OLogit	OLogit

This table shows a set of regressions where the dependent variable is a numerical measure exchange rate regimes (higher values correspond to a more flexible exchange rate regime) and the explanatory variables are OSIN3, the log of GDP per capita (Ln(GDP PC)), public debt as a percentage of GDP (Debt/GDP), external debt over GDP (Ext Debt/GDP), and trade openness (OPEN)

*t* statistics in parentheses

^*^p < 0.10; ^**^p < 0.05; ^***^p < 0.01

The last three columns of Table 5 shows that the preceding results are entirely driven by cross-sectional variation. When we estimate the model including country fixed effects, we do not find any variable that is significantly correlated with the exchange rate regime.

Next, we check whether the preceding results are robust to using the international and domestic indexes of original sin computed using the central government bond dataset of Arslanalp and Tsuda. Results in the first two columns of Table 6 are qualitatively similar to those of columns 2 and 5 of Table 5. This is not surprising, since most of the countries included in the Arslanalp and Tsuda dataset are part of the EMBI index, as noted above. It is, however, reassuring that the two measures of original sin derived using different data sources give similar results when they are applied to a similar sample of countries.

**Table 6:** Original sin and Exchange rate regime (AT data)

	(1)	(2)	(3)	(4)
	Non-residents		Residents	
OSIN AT	-2.460^***^	-0.920	-5.781^***^	17.037^***^
	(-5.55)	(-0.43)	(-6.60)	(3.35)
Ln(GDP PC)	1.880^***^	-0.162	1.702^***^	0.682
	(9.75)	(-0.08)	(8.91)	(0.37)
Ext Debt/GDP	-0.047^***^	-0.027	-0.051^***^	-0.027
	(-8.42)	(-1.21)	(-9.08)	(-1.29)
Debt/GDP	0.018^***^	0.057	0.011^*^	0.038
	(3.06)	(1.19)	(1.89)	(0.62)
Obs	289	198	287	198
R^2^	.16	.03	.17	.21
Country FE	No	Yes	No	Yes
Model	OLogit	OLogit	OLogit	OLogit

This table shows a set of regressions where the dependent variable is a numerical measure of exchange rate regimes (higher values correspond to a more flexible exchange rate regime) and the explanatory variables are the original sin index computed with AT data for non-resident holdings of government bonds (columns 1 and 2) and for holding of government bonds by residents (columns 3 and 4), the log of GDP per capita (Ln(GDP PC)), public debt as a percentage of GDP (Debt/GDP), and external debt over GDP and trade openness Ext Debt/GDP)

*t* statistics in parentheses

^*^p < 0.10; ^**^p < 0.05; ^***^p < 0.01

**Fig. 9:**
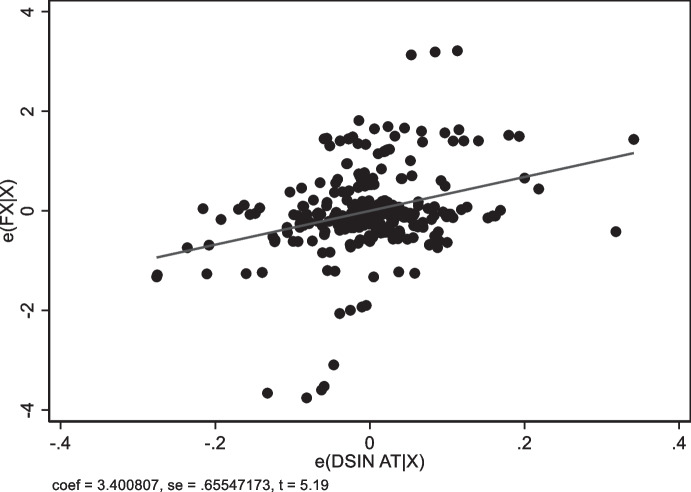
Domestic Original Sin and Exchange Rate Flexibility. The figures plot the partial correlation (controlling for all the variables included in columns 4 of Table 6, but estimated with OLS with Fixed effects) between Domestic Original sin computed with AT data and exchange rate flexibility

The last two columns of Table 6 focus on domestic original sin (the share of foreign currency central government bonds held by residents). Regressions not including country fixed effects are essentially identical to those that measure original sin using non-resident bond holdings (compare columns 1 and 3). However, domestic original sin is strongly correlated with exchange rate flexibility even when we control for country fixed effects. The point estimates indicate that higher values of domestic original sin are associated with more flexible exchange rates. Figure 9 shows the partial correlation between domestic original sin and exchange rate flexibility. While there are some influential observations (Russia, India, Indonesia and Peru), the results are robust to excluding any of these countries.^10^

Finally, we explore the link between original sin and creditworthiness as measured by sovereign ratings. We use a numerical scale that ranges between 1 (selective default) and 18 (AAA) and an ordered logit model.^11^ OSIN is negatively correlated with the likelihood of having a high rating when we include the full sample of countries (column 1 of Table 7) and for countries included in the EMBI index (column 2). This pattern is robust to including country fixed effects (in columns 4 and 5). But the correlation between original sin and credit ratings is not statistically significant for non-EMBI countries (columns 3 and 6). Levels of both public debt and external debt are negatively correlated with credit ratings. This is not unexpected, since credit rating agencies regularly invoke debt ratios as justification for their decisions. However, external debt is not statistically significant in the fixed effects regressions. This suggests that rating agencies are in fact more concerned with other (unspecified) country characteristics. OSIN is not significant for non-EMBI countries, even in the model that does not include country fixed effects (see columns 3–6).

**Table 7:** Original sin and Credit ratings

	(1)	(2)	(3)	(4)	(5)	(6)
OSIN3	-2.834***	-1.378^***^	-0.331	-4.970^***^	-4.465^**^	-4.445
	(-9.13)	(-4.05)	(-0.25)	(-2.64)	(-2.24)	(-0.88)
Ln(GDP PC)	0.995***	1.136^***^	1.171^***^	1.746^***^	2.146^***^	1.146^*^
	(16.36)	(11.06)	(14.18)	(3.66)	(3.02)	(1.87)
Debt/GDP	-0.027***	-0.016^***^	-0.033^***^	-0.065^***^	-0.055^***^	-0.065^***^
	(-14.27)	(-4.66)	(-13.86)	(-7.55)	(-2.70)	(-6.80)
Ext Debt/GDP	-0.004***	-0.008^***^	-0.001	-0.003	-0.011	-0.002
	(-3.49)	(-3.39)	(-1.21)	(-0.65)	(-0.69)	(-0.39)
Obs	1440	641	799	1406	636	770
R^2^	.1	.09	.11	.32	.39	.25
Sample	Panel (annual data)					
Countries	All	EMs	Non-EMs	All	EMs	Non-EMs
Country FE	No	No	No	Yes	Yes	Yes
Model	OLogit	OLogit	OLogit	OLogit	OLogit	OLogit

This table shows a set of regressions where the dependent variable is a numerical measure of credit rating (higher values correspond to higher rating) and the explanatory variables are OSIN3, the log of GDP per capita (Ln(GDP PC)), public debt as a percentage of GDP (Debt/GDP), and external debt over GDP and trade openness Ext Debt/GDP)

*t* statistics in parentheses

^*^p < 0.10; ^**^p < 0.05; ^***^p < 0.01

When we use indexes of original sin computed using the Arslanalp and Tsuda data, we find no significant correlation between international original sin and credit ratings in the model that does not include country fixed effects (column 1 of Table 8). However, the negative relationship between original sin and credit ratings becomes significant when we control for country fixed effects (column 2). As before, we find a positive correlation between GDP per capita and credit ratings and a negative correlation between debt levels and credit ratings.

**Table 8:** Original sin and Credit ratings (AT data)

	(1)	(2)	(3)	(4)
	Non-residents		Residents	
OSIN AT	-2.853	-3.104^***^	-11.98**	-7.093***
	(1.56)	(-7.15)	(2.20)	(9.14)
Ln(GDP PC)	1.257	1.001^***^	1.630	0.694***
	(0.88)	(5.55)	(1.22)	(4.37)
Debt/GDP	-0.066**	-0.431^***^	-0.058	-0.042***
	(2.20)	(-3.15)	(1.26)	(7.22)
Ext Debt/GDP	0.014	-0.002	0.011	0.004
	(0.82)	(-0.46)	(0.79)	(1.01)
Obs	1,005	307	1,003	305
R2	0.17	0.08	0.27	0.12
Country FE	No	Yes	No	Yes
Model	Ologit	Ologit	Ologit	Ologit

This table shows a set of regressions where the dependent variable is a numerical measure of credit rating (higher values correspond to higher rating) and the explanatory variables are the original sin index computed with AT data for non-resident holdings of government bonds (columns 1 and 2) and for holding of government bonds by residents (columns 3 and 4), the log of GDP per capita (Ln(GDP PC)), public debt as a percentage of GDP (Debt/GDP), and external debt over GDP and trade openness Ext Debt/GDP)

*t* statistics in parentheses

^*^p < 0.10; ^**^p < 0.05; ^***^p < 0.01

Turning to domestic original sin, we find that higher levels of original sin always result in lower credit ratings (columns 3 and 4 of Table 8); rating agencies evidently respond negatively when residents insist on holding foreign-currency-denominated debt. In the fixed effects regressions of column 4, we also find the usual positive correlation between income per capita and credit ratings, and the usual a negative correlation between debt levels and credit ratings.

Taken together, updated original sin data based on BIS securities and World Bank data, and the data on the composition of central government debt based on Arslanalp and Tsuda data, all corroborate the earlier results of Eichengreen et al. (2005a, 2007) that original sin is associated with less flexible macroeconomic polices and less creditworthiness.

## Case Studies

Three Latin American countries, Colombia, Peru and Uruguay, made at least some progress at reducing original sin in the course of recent decades. The question is how substantial that progress is in reality. As we show below, Uruguay ended up trading off foreign currency borrowing for indexed (in the case of government bonds) and short-term (in the case of corporates) domestic currency debt. Peru has been more successful at placing long-term, fixed-rate local-currency debt with foreign investors, but has been able to do so only do so by accumulating large amounts of international reserves bearing low interest rates in order to reassure nonresident investors of the stability of the exchange rate and by limiting its external borrowing. In addition, non-resident investors tend to hedge their local-currency exposures using forward contracts, with domestic pension funds taking the other side of the hedge. In other words, it is not clear that Peru has freed itself from original sin in a meaningful sense. And although Colombia has made progress in issuing local-currency bonds, aided by its inclusion in the JPMorgan GB-EM-Global index, international original sin remains significant, as we show later in this section.

**Fig. 10:**
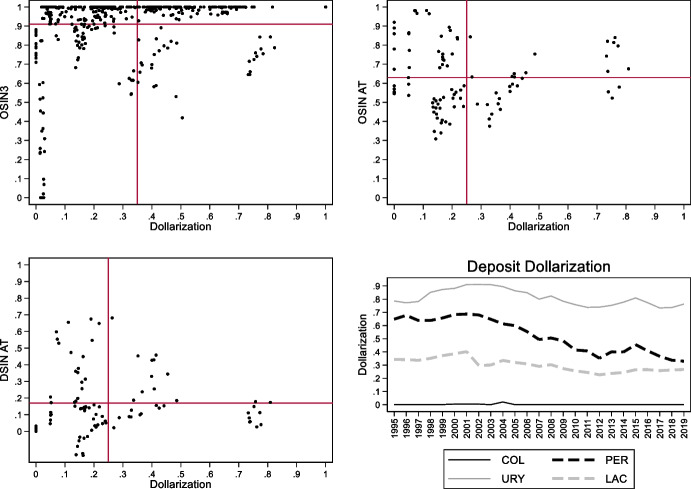
Original Sin and Deposit Dollarization. The first three panels of this figure show the relationship between deposit dollarization (defined as share of foreign currency bank deposits over total bank deposits) and three indexes of original sin (OSIN3 computed with BIS bond data, international original sin computed with AT data, and domestic original sin computed with AT data). The vertical and horizontal lines show the average values of dollarization and original sin. Each point represents a country-year. The bottom right panel shows the evolution of deposit dollarization in Colombia (solid black line), Peru (dashed black line), Uruguay (solid grey line), and for the (unweighted) average of Argentina, Brazil, Chile, Colombia, Mexico, Peru, and Uruguay (dashed grey line)

Uruguay and Peru are further interesting because they have been able to place domestic currency bonds with non-resident investors while also having high levels of domestic dollarization. Ironically, it seems that foreign investors trust the local currency more than residents. To illustrate, the first three panels of Fig. 10 show the relationship between deposit dollarization on the horizontal axis and our three indexes of original sin on the vertical axis.^12^ The vertical and horizontal lines mark average values of dollarization and original sin for all country-years.

**Fig. 11:**
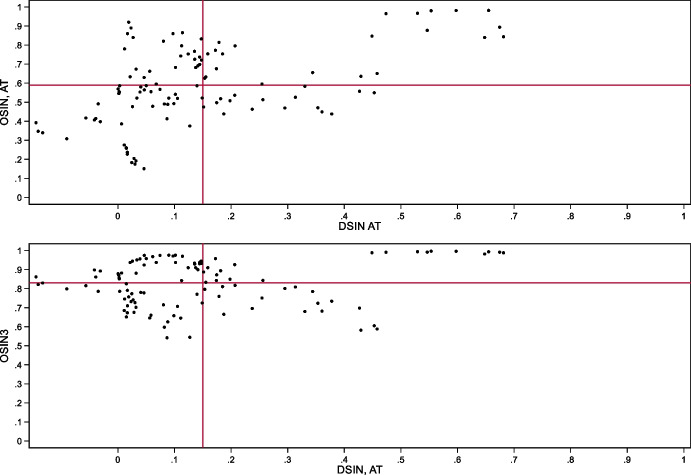
Domestic and International Original Sin. The two panels of this figure show the relationship between domestic original sin (sin computed with AT data) and international original sin computed with AT data (top panel) and BIS bond data (bottom panel). The vertical and horizontal lines show the average values of domestic and international original sin. Each point represents a country-year

The bottom right panel shows the evolution of deposit dollarization in Colombia, Peru, and Uruguay. Peru and Uruguay are the only countries with above average values of domestic dollarization and below average values of international original sin as computed using both BIS and Arslanalp and Tsuda data (top panels of Fig. 10). Peru also has high levels of deposit dollarization and domestic original sin (computed using Arslanalp and Tsuda data), while Uruguay has high levels of dollarization but below average levels of domestic original sin (bottom left panel of Fig. 10; see also Fig. 11 that compares domestic and international original sin).

The increase in the local currency share of Uruguay’s government debt is part of an explicit strategy put in place following the country’s 2002 debt crisis. Key actions aimed at reducing financial fragility arising from currency mismatches included setting up a deposit insurance scheme that privileged peso deposits, reducing reserve requirements for peso deposits, and adopting a public debt management policy focused on increasing the weight of peso debt through the creation of indexed debt instruments and on developing a peso yield curve (Licandro and Licandro 2010). Subsequent initiatives include a coordinated effort by the public debt management office and the Central Bank to deepen peso markets and enhance access by non-residents through Euroclear (Labat and Licandro 2021).

**Fig. 12:**
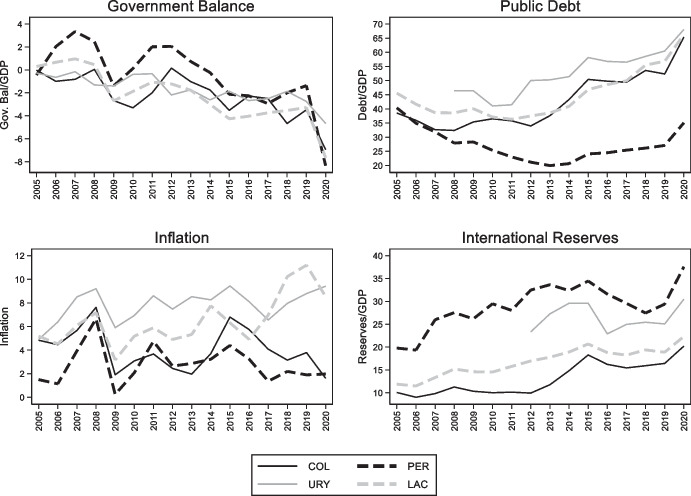
Fundamentals in selected Latin American Economies. The four panels show the evolution of the government budget balance scaled by GDP (top left panel), public debt scaled by GDP (top right panel), inflation (bottom left panel), and international reserves scaled by GDP (bottom right panel) in Colombia (solid black line), Peru (dashed black line), Uruguay (solid grey line), and for the (unweighted) average of Argentina, Brazil, Chile, Colombia, Mexico, Peru, and Uruguay (dashed grey line)

**Fig. 13:**
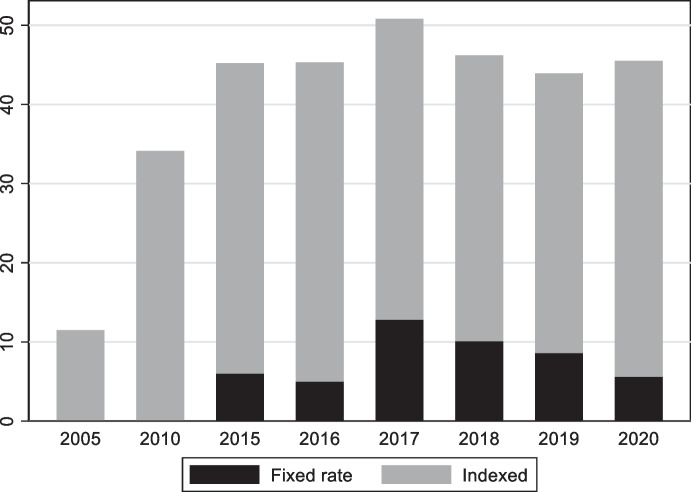
Composition of domestic currency debt in Uruguay. This figure shows the share of domestic currency government debt issued decomposed into inflation and price indexed debt (grey bars) and fixed rate debt (black bars)

These policies have been successful in increasing the share of dollar debt of the public and private sectors, but they have been less successful at improving monetary credibility (see bottom left panel of Fig. 12), reducing deposit dollarization (bottom right panel of Fig. 10), and creating a market for long-term fixed rate peso debt. While the share of local currency debt issued by the Uruguayan government is close to 50%, less than 10% of Uruguay’s debt is issued in domestic currency and with a fixed interest rate (Fig. 13). In other words, these policies ended trading off foreign currency borrowing for indexed (in the case of government bonds) and short-term (in the case of corporates) domestic currency debt. This is an indication that meaningfully solving the original-sin problem requires initiatives to foster local market development but also the maintenance of sound, stable and credit macroeconomic policies, which together are no simple undertaking.

Peru began issuing local currency fixed rate bonds (known as BTP) in 2003. Unlike Uruguay, it has successfully cultivated an international demand for its nominal, long-dated, domestic-currency bonds by putting in place policies that allowed it to benefit from low interest rates in the advanced economies and abundant global liquidity (Rossini et al. 2020) and to accumulate a large stockpile of foreign reserves. A key pillar of this approach is sound macroeconomic policies, which led to fiscal surpluses, a rapid reduction in public debt (in part due to high GDP growth associated with high commodity prices), low inflation, and a large stock of international reserves (Fig. 12). Greater stability of macroeconomic policy is one factor differentiating it from Uruguay. Associated with that stability are surpluses that have facilitated the accumulation of reserves, whose presence works to reassure foreign investors of the stability of the exchange rate, which is a perquisite for their willingness to hold local-currency bonds. But that reserve accumulation comes at a price, since interest rates on dollar and other foreign currency reserves are below those on government debt. Note also that Peru has engaged as much in “abstinence” (limiting new borrowing such that the debt-to-GDP ratio declined significantly) as it has in redeeming itself from original sin. In other words, it is unclear whether, had it engaged in further external borrowing, it could have succeeded in denominating the additional government bonds placed with foreign investors in local currency.

A second pillar of Peru’s approach consists of policies aimed at de-dollarizing the economy and creating a fixed-income market in domestic-currency obligations. In 2013 the central bank initiated a program of credit de-dollarization, imposing higher reserve requirements for foreign currency than domestic currency deposits and adding further reserve requirements when banks’ dollar lending exceeded a set of thresholds fixed by the central bank (Castillo et al. 2016; Contreras et al. 2019; Armas Rivas et al. 2021). Another tool was the creation of a repo facility that allows banks to use their foreign currency reserve to obtain long-term central bank funding in domestic currency, which can then be used to de-dollarize bank loans, either through new loans or by converting old dollar loans (Castillo et al. 2016).

**Fig. 14:**
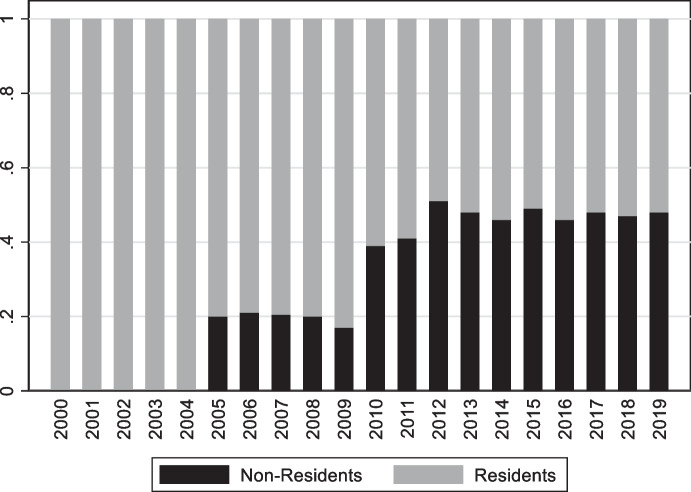
Participation of non-Residents in the Peruvian BTP market. This figure shows the share of non-residents investors (black bars) Peruvian BTP market

With respect to securities, development of the local-currency bond market was fostered by a Market Makers Program implemented by the Ministry of Finance starting in 2003. Its objective was building a public debt market in domestic currency that would then encourage development of a domestic capital market (Rossini et al. 2020). The program focused on issuance of domestic-currency sovereign bonds (the abovementioned BTPs) and promoting active secondary market trading of BTPs. Following its implementation, the share of domestic-currency-denominated bonds in total public debt increased from less than 5% in 2003 to more than 65% in 2020. Over the same period, the average maturity of nominal bonds increased from 1.4 years to over 13 years, with issuances of 30-year bonds starting in 2010 and 40-year bonds since 2014 (Rossini et al. 2020). Nearly 50% of these bonds are now placed with foreign investors (Fig. 14).

While the Market Makers Program was successful in fostering an active market for local currency bonds and attracting foreign investors, it is not obvious that the program enhanced international risk sharing. Participation by foreign investors is heavily influenced by global capital flows and commodity prices (as suggested by the Original Sin Redux hypothesis of Carstens and Shin 2019; Hofmann et al. 2022). Moreover, non-residents investors tend to hedge their local-currency exposure with forward contracts, with domestic pension funds taking the other side of the hedge. In fact, there seems to be a strong negative correlation between the dollar flows created by non-resident investors and domestic pension funds (Rossini et al. 2020). As a result, Peru continues to effectively bear the same currency risk as before.

**Fig. 15:**
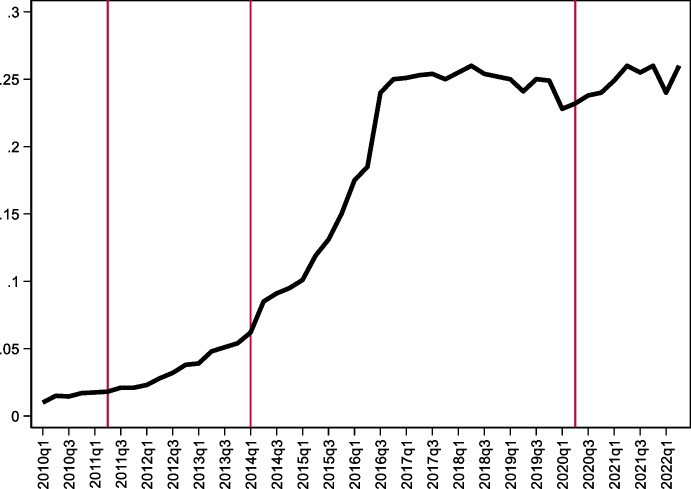
Colombian peso-bonds held by non-residents. This figure plots the share of peso denominated nominal bonds held by non-residents. The first vertical line marks Colombia’s rating upgrade to investment grade, the second vertical line shows the increase of Colombia’s weight in the GB-EM-Global Diversified index, and the third vertical line shows S&P’s downgrade of Colombia’s debt to sub-investment grade

Unlike Peru and Uruguay, Colombia never had high levels of deposit dollarization. Nonetheless, foreign participation in the market for nominal peso denominated bonds remained low (below 5%) until 2014. It then started increasing in 2014, peaking at 25% in 2016 (Fig. 15). Nonetheless, the vast majority of Colombia’s foreign government debt remains either foreign currency denominated or short term (as Fig. 10 shows, original sin remains significant).

The increase in foreign investor participation in Colombia’s domestic-currency bond market was partly driven by good fundamentals (although, somewhat surprisingly, it appears that Colombia’s upgrade to investment grade and subsequent downgrade to junk status did not have a large impact on foreign investors’ participation) and partly driven by the reduction and simplification of taxes on foreign portfolio earning (Vargas et al. 2019). However, Romero et al. (2021) suggest that the key driver for the increase in non-residents participation in the Colombian bond market was JPMorgan’s announcement in March 2014 that Colombia's weight in an index tracking local currency bonds issued by emerging markets (GB-EM-Global Diversified) would increase from 3.9% to 8%.^13^ In 2014, funds that tracked that index amounted to about $220 billion. The increase in weigh was anticipated to lead to inflows of approximately $9 billion (4% of 220) which, according to Romero et al. (2021), was close to the actual inflow. Consistent with this observation, mutual funds (including tracking funds) are the single most important class of foreign investors in Colombian government local-currency bonds (Murcia 2022).

While Colombia’s increased weight in the index led to large inflows, successive reduction in that weight does not appear to have had a major negative impact. The fact that reductions of the weight and the 2021 rating downgrade did not lead to a sudden collapse of foreign investors participation suggest that there is some stickiness in foreign investors participation in local currency markets.

As in other countries, the increased participation of foreign investors in local currency bonds markets required the central to invest in additional international reserves (Vargas et al. 2019). This costly investment was required to ensure that the authorities had sufficient dollar liquidity to intervene in the markets and avoid excessive, disruptive exchange-rate fluctuations if those foreign investors took flight. In addition, an adequate buffer of international reserves was needed to guarantee sufficient stability of the exchange rate for those foreign investors to enter in the first place.

An interesting case outside Latin America is South Africa. South Africa has the largest financial sector and domestic bond market on the African continent. It is one of the few countries in Sub-Saharan Africa with a substantial presence of foreign investors in the local bond market (Essers et al. 2016).

Foreign investor participation in the market for offshore rand-denominated bonds dates back to 1997 when the South African government started issuing bonds in the Euro-Rand market. However, the largest issuers of rand-denominated bonds in the offshore market are not South African nationals. Rather, foreign investors interested in rand bonds issued by the South African government tend to buy them in the onshore market.

BIS data on international bond issuance show that *OSIN3* is substantially lower than *OSIN1*. Our data indicate that international issuance of rand-denominated bonds by South African nationals is often smaller than rand issuance by nationals of other countries (including international organizations). Most of these bonds are issued by international organizations or by domestic financial institutions based in advanced economies (especially in Germany and the Netherlands; see Table 9).

**Table 9:** Share of international rand bond issuances by non-South African Nationals

	**Corporates**	**Fin. Inst.**	**Int.Org**	**Pub. Sect.**	**Total**
**Period 1995–2010**					
Australia	0.00%	0.06%	N/A	0.00%	0.06%
Finland	0.00%	0.79%	N/A	0.10%	0.89%
France	0.00%	0.37%	N/A	0.00%	0.37%
Germany	0.97%	11.84%	N/A	0.64%	13.45%
Int. org	N/A	N/A	63.15%	N/A	63.15%
Italy	0.00%	1.28%	N/A	0.00%	1.28%
Japan	0.00%	1.97%	N/A	0.00%	1.97%
Netherlands	0.00%	8.37%	N/A	0.00%	8.37%
Norway	0.00%	1.44%	N/A	0.03%	1.48%
Saudi Arabia	0.00%	0.11%	N/A	0.98%	1.09%
Sweden	0.00%	2.88%	N/A	0.14%	3.02%
Switzerland	0.00%	0.23%	N/A	0.00%	0.23%
UK	0.00%	1.52%	N/A	0.00%	1.52%
USA	0.19%	1.37%	N/A	0.00%	1.56%
Oth. Countries	0.00%	0.76%	N/A	0.80%	1.56%
**Total**	**1.17%**	**33.00%**	**63.15%**	**2.68%**	**100.00%**
**Period 2011–2021**					
Australia	0.00%	1.03%	N/A		1.03%
Finland	0.00%	1.21%	N/A	0.69%	1.89%
France	0.00%	3.14%	N/A	0.04%	3.18%
Germany	0.07%	6.84%	N/A	0.84%	7.75%
Int. org	N/A	N/A	53.78%	N/A	N/A
Italy	0.00%	0.00%	N/A	0.00%	0.00%
Japan	0.00%	2.09%	N/A	0.00%	2.09%
Netherlands	0.00%	10.36%	N/A	0.11%	10.47%
Norway	0.00%	0.73%	N/A	0.67%	1.39%
Saudi Arabia	0.00%	0.47%	N/A	0.92%	1.39%
Sweden	0.00%	2.09%	N/A	0.81%	2.90%
Switzerland	0.00%	3.53%	N/A	0.00%	3.53%
UK	0.00%	4.80%	N/A	0.00%	4.81%
USA	0.00%	3.31%	N/A	0.00%	3.31%
Oth. Countries	0.00%	2.08%	N/A	0.39%	2.48%
**Total**	**0.07%**	**41.68%**	**53.78%**	**4.47%**	**100.00%**

Public sector includes state-owned corporations and financial institutions

**Fig. 16:**
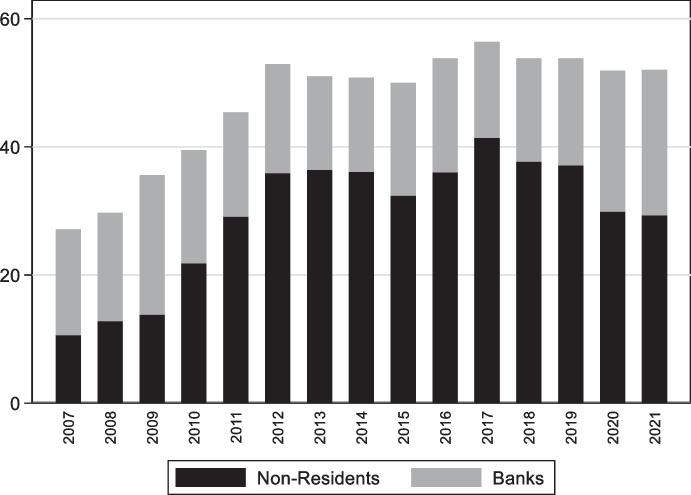
Holders of South African domestic government bonds. This figure shows the share of domestic currency local government bonds held by non-residents (black bars) and South African Banks (grey bars). Other large players in the market (not reported in the graph) are pension funds which hold between 20 and 30% of outstanding domestics bonds and insurance companies and other financial institutions which hold between 20 and 25% of domestic government bonds

**Fig. 17:**
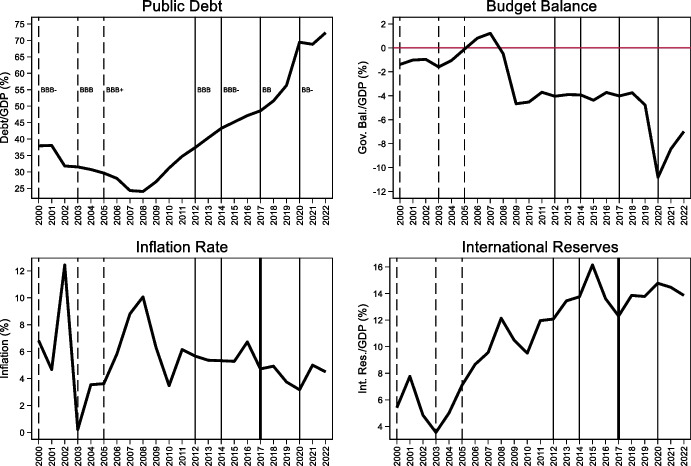
South Africa Fundamentals. The four panels plot the evolution of the debt-to-GDP ratio, government balance to GDP ratio, inflation, and international-reserves-to GDP. The dashed vertical line mark rating upgrades (to BBB- in 2000, to BBB in 2003, and to BBB + in 2005) and the solid vertical lines show rating downgrades (to BBB in 2012, BBB- in 2014, BB + and then BB in 2017, BB_ in 2020), the think vertical line shows the year in which Suth Africa received two downgrades and lost its investment grade status

As mentioned, non-residents play an important role in the onshore market for fixed-rate government bonds (Fig. 16). The share of foreign investors in this market increased from about 10% in 2007 to a peak of 41% in 2017. That this increased participation by non-residents occurred in the presence of deteriorating fundamentals and a series of rating downgrades (Fig. 17) suggests that it was driven by favorable global financial conditions. This foreign investor participation in the offshore market allowed South Africa to reduce the share of foreign currency government bonds from about 16% in 2008 to 7% in 2018 (the share of foreign currency bonds is now just above 10%) and to extend the average maturity of domestic government fixed rate debt.

**Fig. 18:**
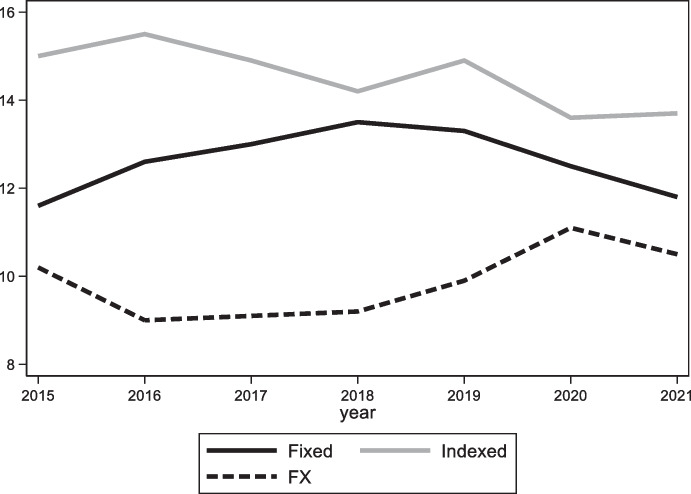
Average maturity of South African Government Bonds. The solid black line plots the average maturity of Fixed rate South African government bonds and bills denominated in domestic currency, the solid gray line plots the average maturity of inflation indexed South African government bonds denominated in domestic currency, and the dashed gray line plots the average maturity of South African government bonds denominated in foreign currency

Foreign participation in the local government bond market then declined once South Africa lost its investment-grade status in 2017.^14^ The 2017 downgrade was also followed by a reduction in the average maturity of local currency fixed rate government bonds (Fig. 18). These recent trends notwithstanding, foreign investors remain the largest holders of local currency fixed rate bonds and the average maturity of these bonds is still well above ten years (up from about 8 years in 2008).

**Fig. 19:**
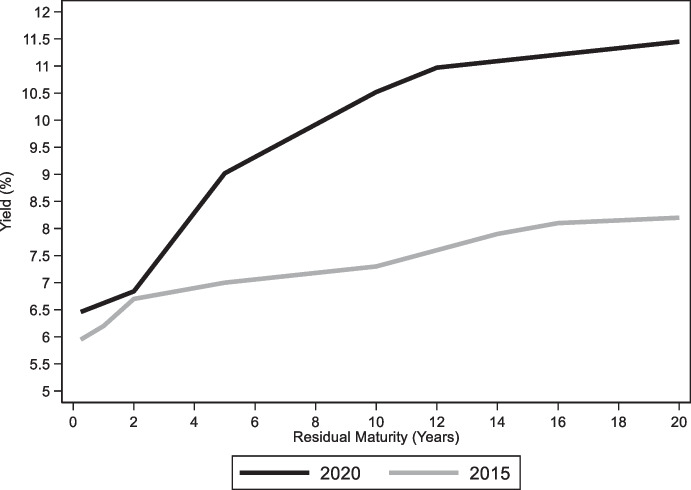
Yield curves for South African Fixed Rate Domestic Currency. This figure plots the yield curves for south African Domestic Currency government bonds in 2015 (grey line) and 2022 (black line)

Although reliance on rand borrowing allowed the South African government to limit risks associated with currency mismatches, it also had a cost in terms of high ex-ante interest rates. This is especially the case for long-dated local currency bonds, because the deterioration in fundamentals illustrated in Fig. 17 was associated with a steeping of the local currency yield curve. The yield differential between 3-year and 10-year bonds went from about 100bps in 2015 to over 500bps in 2020 (Fig. 19). The increase in long term rates, together with an increase of the spread of foreign currency bonds, had serious fiscal consequences in that it brought interest payments from about 7% of government revenues in 2008 to 12% of government revenues in 2019. (Hausmann et al. 2022).

There are two lessons of this South African experience. The first lesson relates to the role of the offshore market for local currency bonds. Currently, there are few emerging market countries (especially in the African continent) with an actual or potential market size comparable to South Africa. It is thus unlikely that many international investors will pay the fixed costs necessary to enter these markets. The alternative is to follow the indirect route: local currency issuance by both nationals and highly rated non-nationals could create an initial appetite for bonds denominated in the currencies of these emerging markets which could then lead to a direct presence in the onshore market.

The second lesson has to do with tradeoffs. While South Africa has been successful attracting foreign investors to its market, when fundamental deteriorated large domestic currency borrowing led to high interest rates with a large fiscal cost.

## Low-Income Economies and Policy Initiatives^15^

Although some progress has been in middle-income economies, low-income economies continue to have very limited access to external funding in local currency. Virtually all domestic currency funding is locally sourced, with negative implications for access to credit by the private sector. Local funding is useless for countries that need external funds to close their financing gaps.

In addition to that, almost all official lending from the World Bank, IDA, and other multilateral development banks is denominated in foreign currencies. There are good reasons why international financial institutions (IFIs) lend in dollars. Prudential practices do not allow them to assume currency risk, so loans in domestic currency must be backed by domestic currency borrowing. In theory, IFIs should be able to borrow at cheaper rates than their clients, who endure higher credit risk, but in practice this is rarely the case. Multilateral development banks seem to have a comparative advantage in borrowing and lending in foreign currency, where the only thing that matters is credit risk.

While multilateral financial institutions do not appear to have an advantage in issuing local currency bonds of their borrowing countries, they may have an advantage in developing new indexed instruments. Eichengreen et al. (2007) and Eichengreen and Hausmann (2005) suggest that the World Bank and other multilateral development banks could issue bonds denominated in a real (i.e., indexed to inflation) emerging market currency index and use the proceeds to extend local currency inflation-indexed loans to their clients. Such a policy would have two advantages: (i) it would allow the multilaterals to lend in local currency (albeit indexed to prices) without taking a currency risk, and (ii) it could create a market that could then be tapped by other types of issuers. Although Eichengreen and Hausmann (2005) showed that a local currency indexed bond would have desirable risk-return features, and although the proposal has been discussed in several high-level fora, multilateral financial institutions have not yet made actual moves in this direction.

Hausmann and Rigobon (2003) put forward a similar proposal targeted at IDA (and, more generally, concessional) lending. These authors start by recognizing that while the IBRD window of the World Bank lends in dollars because it issues most of its debt in dollars, the IDA window, mostly funded with fiscal transfers and retained World Bank Group earnings, could lend in any currency. They suggest that IDA should lend in inflation-indexed domestic currency, and show that this would not have large budgetary implications. They suggest such a portfolio could in fact generate higher returns than a dollar denominated portfolio.^16^ Bachiocchi and Missale (2015) conduct a quantitative analysis using a VAR model and confirm that local currency lending would be beneficial for IDA. Given all these policy proposal, it is somewhat puzzling that the multilaterals have not experimented with possible ways to reduce currency risk associated with their lending.

Kapoor et al. (2021) suggest that the exposure of developing country borrowers to currency risks could be mitigated by the creation of a multilateral International Currency Fund (ICF). They envision the ICF as a multilateral agency with preferred creditor status and the mandate of supporting the creation of currency risk markets by providing two-way markets in currencies and tenors where private markets do not yet exist. In their view, the ICF will act as a catalyst and crowd the private sector that can then bring additional hedging capacity and diversity and will ultimately promote the development of domestic currency bond market. On the basis of the experience of TCX, Kapoor et al. (2021) estimate that the fund that they envision could easily offset up to 75% of its exposure and that the residual risk could be managed though diversification.

Perry (2009) moves one step further and suggests that multilateral development banks should retain currency risk. He argues that doing so would not have negative implications for institutions such as the World Bank that can pool currency risk across countries.

With specific reference to regional development banks, an alternative would be to offload the currency risk to funds that are able to achieve global diversification. For instance, TCX (The Currency Exchange Fund) hedging would allow regional development banks to achieve the benefit of global diversification or also allow an institution like IDA to experiment with local currency lending in a limited subset of countries.^17^ Perry (2009) describes a possible swap with TCX as follows:

TCX would accept foreign exchange exposures on transactions originated primarily by its customers (for the first three years only by its shareholders) in hard currencies, by offering swaps and forwards to convert them into domestic currencies for the beneficiaries at the same maturities. Originating customers would retain the credit risk, so that TCX would retain only the currency risk, and though it plans to diversify some away through existing derivative markets, it expects to achieve most risk diversification through its global pooling. TCX estimates that its global fund of developing country domestic currencies can achieve, on average, a 75 percent risk reduction in comparison with a single currency risk. Regional development banks and other investors would have guaranteed access for about three to four times their equity investment in TCX (some will join through deeply subordinated debt instruments). [...] Although of a modest initial size, TCX has the potential to achieve a significant impact in small- and medium-sized countries where local currency markets are small and essentially short-term. (Perry 2009 p. 45-46)

As predicted by Perry, TCX has indeed grown rapidly. Since its inception, TCX has provided currency-hedging instruments for more than 3500 private sector external lending operations. In 2020, TCX targeted a total volume of about US$3 billion in exotic currency swaps. It raised additional capital to increase its total swap portfolio to more than 7 billion in 2022 so as to act as market maker in the currencies of low-income countries.

One obstacle to this kind of market creation relates to the fact that low-income countries have limited debt management capacity. Their debt managers may not fully appreciate the costs and benefits of local currency instruments with an embedded insurance component (Paesani and Piga 2010). They may be afraid of being duped into issuing complex and costly instruments by slick investment bankers. One policy response is to strengthen local debt management capacity, so that debt managers are better equipped to understand and evaluate the cost/risk tradeoffs of different borrowing options. In any case, limited capacity should not be an obstacle when dealing with multilateral financial institutions that do not have a profit motive and that could offer just one type (or a limited menu) of domestic currency instruments with transparent rules, possibly (in the case of IDA), at subsidized rates.^18^

Even in the absence of distortions, a fair currency premium would typically incorporate the possibility of a sharp devaluation in the future. This is analogous to buying insurance that, by its very nature, implies a premium that must be paid during good times. While a forward-looking benevolent policymaker would see that the premium, when appropriately priced, exactly compensates for currency risk, and, in the presence of risk aversion, would opt for the safer local currency debt, myopic policymakers who only care about the present would disregard negative events that may materialize when they are no longer in office. If, as usual, the probability of a currency adjustment increases with the time horizon, myopic policymakers would find the premium expensive relative to short-term risk, and opt for foreign currency debt which would commands a lower interest rate but leave future governments exposed to currency risk. Again, the multilateral financial institutions could play a role in mitigating these political failures by clearly explaining to policymakers and to the public the insurance benefits of local currency debt. Therefore, disseminating debt management best practices across member countries would be a necessary condition for the development of local currency instruments.

## Data Availability

Some of the data used in the current study were obtained on a confidential basis and cannot be shared. All other data are available from the sources quoted in the paper.

## Footnotes

1 Here emerging markets is used as shorthand for emerging markets and developing countries or emerging markets and low-income countries. We draw the distinction more carefully below.

2 Contributors to the literature on “debt intolerance” (Reinhart et al. 2003) suggested that this lack of credibility was the consequence of a history of inflation and default. But this only moved the puzzle back in time; why emerging markets should have had more fraught histories of inflation and default went unexplained. So too was the mechanism responsible for persistence – for the fact that these past histories continued to limit modern economies’ ability to manage debt. Others (e.g. Giordano and Tommasino 2011) suggested that emerging markets’ political and monetary institutions were at fault. But why those institutions were so impervious to change again begged the question.

3 Benetrix et al. (2019) caution that BIS data on cross-border liabilities sometimes fail to fully capture foreign investor holdings of domestic currency denominated bonds.

4 Eichengreen et al. (2005a, b, 2007) also built a third measure called OSIN 2 which also used data on bank loans. However, this measure was problematic because of lack of detailed data on the currency composition of bank loans.

5 The reader will infer that in these earlier papers we also considered yet another index, OSIN 2, which for reasons of brevity is omitted here. Nonetheless, for consistency with that earlier paper we keep the variable names the same as before.

6 These countries are Bulgaria, Ghana, Hungary, India, Indonesia, Kazakhstan, Malaysia, Mexico, Poland, Romania, Russia, and South Africa).

7 Calculating a lower bound work against our argument, which is that original sin remains an important factor for a range of developing economies.

8 China, Russia, and South Africa all countries with values of OSIN3 which are well below OSIN 1.

9 India is notorious for force-feeding domestic-currency-denominated government securities to its banks, which are in turn limited in their ability to lend to the private sector for purposes related to economic development (Gandhi 2016).

10 However, they are not robust to the joint exclusion of Peru and Indonesia (when we drop these two countries the coefficient remains positive but p value associated with domestic original sin goes to 0.11).

11 Whenever available, we use S&P ratings, but for countries for which we do not have S&P rating, we use Moody’s or Fitch rating if they are available.

12 Deposit dollarization is defined as the share of domestic bank deposits in foreign currency over total bank deposits. The three indexes of original sin are OSIN3 computed with BIS bond data, international original sin computed with Arslanalp and Tsuda data, and domestic original sin computed with Arslanalp and Tsuda data.

13 For a discussion of benchmark driven investment in emerging markets see Arslanalp et al. (2020)

14 This decrease in foreign investors participation was compensated by an increase in the share of local currency government bonds held by domestic banks (government bonds now represent more than 15% of domestic bank assets, Hausmann et al. 2022).

15 This section draws from Panizza and Taddei (2020).

16 Hausmann and Rigobon (2003) argued that switching to inflation indexed local currency would increase likelihood of repayment because the debt burden would be larger in good periods and smaller in bad periods, improve risk management for low-income countries and only have a minor overall impact the IDA portfolio’s dollar value. dePlaa and Yi (2005) find that switching to inflation index local currency would indeed generate benefits for borrowing countries as it would greatly reduce the sensitivity of the debt-to-GDP ratio to currency depreciations (according to dePlaa and Yi’s, 2005, estimates a negative shock to the exchange rate would increase the debt-to-GDP ratio by 0.39 standard deviations under current IDA practices and by 0.07 standard deviations under the Hausmann and Rigobon proposal). However, dePlaa and Yi (2005) find no benefit (but also no cost) for IDA’s portfolio diversification. While economic theory predicts that the real exchange rate od low income countries should appreciate over time (this is the Balassa-Sameulson effect), dePlaa and Yi (2005) show that over 1985-2005 the real exchange rate of IDA countries depreciated and that an inflation indexed local currency lending program would have reduced reflows by about $1 billion. It is worth noting that this is not a large amount considering that over the same period IDA replenishments amounted to more than $100 billion.

17 TCX is a fund that provides hedges which allow to manage risk in more than 70 currencies and maturities which are not normally covered by private institutions. TCX was founded in 2007 by a group of development finance institutions (DFIs) with also initial funding by the Dutch and German government. Its 22 shareholders include bilateral and multilateral development agencies.

18 Adverse selection can also be a problem. This is less of a problem with official lenders that have a cooperative nature and can offer subsidized loans in a take-it or leave offer.

## References

[CR1] Aizenman J, Jinjarak Y, Park D, Zheng H (2020) Good-Bye original sin, hello risk on-off, financial fragility and financial crises. NBER Working Paper no. 27030

[CR2] Alfaro L, Calani M, Verala L (2021). Currency Hedging in Emerging Markets: Managing Cash Flow Exposure.

[CR3] Arslanalp MS, Tsuda MT (2014) Tracking global demand for emerging market sovereign debt. IMF Working Paper 14/39

[CR4] Arslanalp S, Drakopoulos D, Goel R, Koepke R (2020) Benchmark-driven investments in emerging market bond markets: taking stock. IMF Working Paper no. 20/192

[CR5] Bacchiocchi E, Missale A (2015). Multilateral indexed loans and debt sustainability. Oxf Rev Econ Policy.

[CR6] Ballard-Rosa C, Mosley L, Welhausen R (2022). Coming to Terms: The Politics of Sovereign Bond Denomination. Int Organ.

[CR7] Bassetto M, Galli C (2019). Is Inflation Default? The Role of Information in Debt Crises. American Economic Review.

[CR8] Benetrix AS, Gautam D, Juvenal L, Schmitz M (2019) Cross-border currency exposures: new evidence based on an enhanced data set. IMF Working Paper no. 19/299

[CR9] Bertaut CC, Bruno V, Shin HS (2020) Original sin redux, manuscript. Bank for International Settlements

[CR10] Borensztein ME, Jeanne MO, Mauro MP, Zettelmeyer MJ, Chamon MM (2004) Sovereign debt structure for crisis prevention. Occasional Paper no. 237, Washington, D.C.: IMF

[CR11] Borensztein E, Cowan K, Eichengreen B, Panizza U (2008). Bond Markets in Latin America: On the Verge of a Big Bang?.

[CR12] Burger JD, Warnock FE, Warnock VC (2012) Emerging local currency bond

[CR13] Calvo GA (1988) Servicing the public debt: the role of expectations. Am Econ Rev Am Econ Assoc 78(4):647–661

[CR14] Calvo GA, Reinhart CM (2002) Fear of floating. Quarter J Econ v107(2):379–408

[CR15] Carstens A, Shin HS (2019) Emerging markets aren’t out of the woods yet. Foreign Affairs

[CR16] Castillo P, Vega H, Serrano E, Burga C (2016) De-dollarization of credit in Peru: the role of unconventional monetary policy tools. Central Bank of Peru, Documentis de Trabajo N 2016–002

[CR17] Contreras A, Gondo R, Pérez F, Oré E (2019) Assessing the impact of credit dedollarization measures in Peru. Central Bank of Peru, Documentis de Trabajo N 2019–005

[CR18] De Grauwe P (2012) The governance of a fragile eurozone. Aust Econ Rev 45:255–268

[CR19] Dell’Erba S, Hausmann R, Panizza U (2013). Debt levels, debt composition, and sovereign spreads in emerging and advanced economies. Oxf Rev Econ Policy.

[CR20] dePlaa A, Yi S (2005) Local currency-denominated borrowing: do the benefits outweigh the costs? unpublished. The World Bank

[CR21] Du W, Schreger J (2017). Sovereign Risk, Currency Risk, and Corporate Balance Sheets.

[CR22] Eichengreen B, Hausmann R (1999) Exchange rates and financial stability. In Federal Reserve Bank of Kansas City, New Challenges for Monetary Policy, Kansas City: Federal Reserve Bank of Kansas City, pp 319–367

[CR23] Eichengreen B, Hausmann R, Panizza U (2007) Currency mismatches, debt intolerance, and the original sin: why they are not the same and why it matters. nber chapters. In: Capital Controls and Capital Flows in Emerging Economies: Policies, Practices, and Consequences, pages 121–170 National Bureau of Economic Research, Inc

[CR24] Eichengreen B, Hausmann R (2005) The road to redemption. In Eichengreen et al. Other People’s Money - Debt Denomination and Financial Instability in Emerging Market Economies, University of Chicago Press, Chicago and London

[CR25] Eichengreen B, Hausmann R, Panizza U (2005a) The pain of original sin. In Eichengreen et al. Other People’s Money - Debt Denomination and Financial Instability in Emerging Market Economies, University of Chicago Press, Chicago and London

[CR26] Eichengreen B, Hausmann R, Panizza U (2005b) The mistery of original sin. In Eichengreen et al. Other People’s Money - Debt Denomination and Financial Instability in Emerging Market Economies, University of Chicago Press, Chicago and London

[CR27] Engel C, Park J (2018) Debauchery and original sin: the currency composition of sovereign debt. NBER Working Paper no. 24671

[CR28] Essers D, Blommestein HJ, Cassimon D, Flores PI (2016). Local Currency Bond Market Development in Sub-Saharan Africa: A Stock-Taking Exercise and Analysis of Key Drivers. Emerg Mark Financ Trade.

[CR29] Financial Stability Board (2022) US dollar funding and emerging market economy vulnerabilities. Basel: Financial Stability Board

[CR30] Flandreau M, Sussman N (2005) Old sins: exchange rate clauses and european foreign lending in the 19th century. In Eichengreen et al. Other People’s Money - Debt Denomination and Financial Instability in Emerging Market Economies, University of Chicago Press, Chicago and London

[CR31] Baumann Fonay I (2022) The effects of foreign investors’ holdings on the local currency sovereign bond markets in Latin America. Technical Note no. IDB-TN-2451, Washington, D.C.: Interamerican Development Bank

[CR32] Gandhi R (2016) Challenges in developing the bond market in BRICS. Paper presented to the seminar on Challenges in Developing the Bond Market in BRICS. Mumbai

[CR33] Gegenfurtner D (2021) The Causes of original sin: an empirical investigation of emerging markets and developing countries. Working Paper no. 174/2021, Berlin: Institute for International Political Economy, Berlin School of Economics and Law

[CR34] Goldstein M, Turner P (2004). Controlling Currency Mismatches in Emerging Markets.

[CR35] Hale GB, Jones PC, Spiegel MM (2020) Home currency issuance in international bond markets. J Int Econ 122

[CR36] Han B (2022) Original sin dissipation and currency exposures in emerging markets. Manuscript, Bank of Korea

[CR37] Hausmann R, Rigobon R (2003). IDA in UF: on the benefits of changing the currency denomination of concessional lending to low-income countries.

[CR38] Hausmann R, Panizza U, Stein E (2001) Why do countries float the way they float? J Dev Econ, Elsevier 66(2):387–414

[CR39] Hausmann R, Panizza U (2011) Redemption or abstinence? Original sin, currency mismatches and counter cyclical policies in the new millennium. J Global Dev, De Gruyter 2(1):1–35

[CR40] Hausmann R, Sturzenegger F, Goldstein P, Muci F, Barrios D (2022) Macroeconomic risks after a decade of microeconomic turbulence: South Africa 2007–2020. Harvard CID Faculty Working Paper No. 404

[CR41] Hofmann B, Shim I, Shin HS (2020) Emerging market economy exchange rates and local currency bond markets amid the Covid-19 pandemic. BIS Bullet

[CR42] Hofmann B, Shim I, Shin HS (2021) Original sin redux and policy responses in EMEs during the Covid-19 pandemic. In S. Djankov and U. Panizza (eds) *COVID-19 in Developing Economies*, CEPR Press, London

[CR43] Hofmann B, Patel N, Wu SP (2022) Original sin redux: a model-based evaluation. BIS Working Paper no. 1004, Basel: Bank for International Settlements

[CR44] Ilzetzki E, Reinhart CM, Rogoff KS (2019). Exchange Arrangements Entering the 21st Century: Which Anchor Will Hold?. Quart J Econ.

[CR45] International Monetary Fund and World Bank (2016) Development of local currency bond markets: overview of recent developments and key themes. Staff Note for the G20

[CR46] Kapoor S, Hirschhofer H, Kapoor D, Klieterp N (2021) A multilateral solution to hedging currency risk in developing country finance. NIFTYS Policymaker notes series “Big Solutions for Big Problems”

[CR47] Lee A (2022) Why do emerging economies borrow in foreign currency? the role of exchange rate risk. Manuscript, University of Wisconsin-Madison

[CR48] Labat D, Licandro G (2021) Towards a quality currency. Central Bank of Uruguay, Documento de Trabajo N. 0005–2021

[CR49] Licandro G, Licandro JA (2010) Fragilidad Financiera y Dolarización: ¿Cuánto se Avanzó? Reconstruir el Mercado en Pesos. Desdolarización y Fragilidad Financiera: Avances y Agenda. Pendiente. UCU. Montevideo

[CR50] Mehl A, Reynaud JP (2005) The determinants of ‘domestic’ original sin in emerging market economics. ECB Working Paper no. 560

[CR51] Mizen P, Packer F, Remolona E, Tsoukas S (2021) Original sin in corporate finance: new evidence from asian bond issuers in onshore and offshore markets. J Int Money Finance 119

[CR52] Murcia A (2022). “Some Facts on ‘Original Sin’ in Colombia.

[CR53] Ottonello P, Perez DJ (2019a) The currency composition of sovereign debt. AEJ: Macroeconom 11:174–208

[CR54] Ottonello P, Perez DJ (2019). The Currency Composition of Sovereign Debt. Am Econ J Macroecon.

[CR55] Paesani P, Piga G (2010) Government debt servicing and macroeconomic risk in emerging economies: lessons learned and recommendations. UNCTAD

[CR56] Panizza U, Taddei F (2020) Local currency denominated sovereign loans - a portfolio approach to tackle moral hazard and provide insurance. IHEID Working Papers 09–2020

[CR57] Perry G (2009). Beyond Lending.

[CR58] Reinhart C, Rogoff K, Savastano M (2003). Debt intolerance. Brook Pap Econ Act.

[CR59] Rivas AA, Misaico ZQ (2021) Dollarization dynamics and de-dollarization policies ineru. Central Bank of Argentina. Ensayos Económicos 77

[CR60] Romero JV, Vargas H, Cardozo P, Murcia A (2021) How foreign participation in the colombian local public debt market has influenced domestic financial conditions. Latin Am J Central Bank 2(4)

[CR61] Rossini R, Montoro C, Luna M (2020) Financial market development and monetary policy: the Peruvian experience. BIS Paper no. 113, Basel: Bank for International Settlements

[CR62] Schmittmann J (2010) Currency hedging for international portfolios. IMF Working Paper no. 10/151

[CR63] Shin HS (2021) Overcoming ‘original sin’ to secure policy space. Manuscript, Bank for International Settlements

[CR64] Shin HS, von Peter G (2022) Overcoming original sin. J Global Dev (forthcoming)

[CR65] Tirole J (2003). Inefficient foreign borrowing: A dual and common-agency perspective. American Economic Review.

[CR66] Vargas H, Cardozo P, Villamizar M (2019) International reserve policy and the effectiveness of sterilized fx intervention in Colombia. BIS Paper no. 104, Basel: Bank for International Settlements

[CR67] Venkatesh H, Hiremath GS (2021) the resurgence of currency mismatches: emerging market economies are not out of the woods yet. Int Econ Econ Policy 10.1007/s10368-021-00500-x PMC8243064 40477465

